# Follicular dendritic cell differentiation is associated with distinct synovial pathotype signatures in rheumatoid arthritis

**DOI:** 10.3389/fmed.2022.1013660

**Published:** 2022-11-16

**Authors:** Mohey Eldin M. El Shikh, Riham El Sayed, Nesreen Amer Ramadan Aly, Edoardo Prediletto, Rebecca Hands, Liliane Fossati-Jimack, Michele Bombardieri, Myles J. Lewis, Costantino Pitzalis

**Affiliations:** Centre for Experimental Medicine and Rheumatology, William Harvey Research Institute, Barts and the London School of Medicine and Dentistry, Queen Mary University of London, London, United Kingdom

**Keywords:** rheumatoid arthritis, synovial pathotypes, follicular dendritic cells (FDCs), platelet-derived growth factor (PDGF), tumor necrosis factor-α (TNF-α), ectopic lymphoid-like structures (ELSs), pericyte and perivascular, germinal center (GC)

## Abstract

Follicular dendritic cells (FDCs) fundamentally contribute to the formation of synovial ectopic lymphoid-like structures in rheumatoid arthritis (RA) which is associated with poor clinical prognosis. Despite this critical role, regulation of FDC development in the RA synovium and its correlation with synovial pathotype differentiation remained largely unknown. Here, we demonstrate that CNA.42^+^ FDCs distinctively express the pericyte/fibroblast-associated markers PDGFR-β, NG2, and Thy-1 in the synovial perivascular space but not in established follicles. In addition, synovial RNA-Seq analysis revealed that expression of the perivascular FDC markers was strongly correlated with PDGF-BB and fibroid synovitis, whereas TNF-α/LT-β was significantly associated with lymphoid synovitis and expression of CR1, CR2, and FcγRIIB characteristic of mature FDCs in lymphoid follicles. Moreover, PDGF-BB induced CNA.42^+^ FDC differentiation and CXCL13 secretion from NG2^+^ synovial pericytes, and together with TNF-α/LT-β conversely regulated early and late FDC differentiation genes in unsorted RA synovial fibroblasts (RASF) and this was confirmed in flow sorted stromal cell subsets. Furthermore, RASF TNF-αR expression was upregulated by TNF-α/LT-β and PDGF-BB; and TNF-α/LT-β-activated RASF retained ICs and induced B cell activation in *in vitro* germinal center reactions typical of FDCs. Additionally, FDCs trapped peptidyl citrulline, and strongly correlated with IL-6 expression, and plasma cell, B cell, and T cell infiltration of the RA synovium. Moreover, synovial FDCs were significantly associated with RA disease activity and radiographic features of tissue damage. To the best of our knowledge, this is the first report describing the reciprocal interaction between PDGF-BB and TNF-α/LT-β in synovial FDC development and evolution of RA histological pathotypes. Selective targeting of this interplay could inhibit FDC differentiation and potentially ameliorate RA in clinically severe and drug-resistant patients.

## Introduction

Follicular dendritic cells (FDCs) critically support the structure and function of ectopic lymphoid-like structures (ELS) that develop in the target tissues of chronic inflammatory autoimmune disorders including rheumatoid arthritis (RA). We have shown that ELSs are present in at least 31% of the RA synovial tissues; 28% of them are FDC^+^ corresponding to 8% of the total RA cohort. Moreover, our studies of the molecular architecture of ELSs in RA provided conclusive evidence that, in the presence of FDCs, ELSs evolve to fully functional germinal centers (GCs) where antigen-specific plasma cell differentiation, affinity maturation, activation-induced cytidine deaminase (AID)-mediated class switch recombination (CSR) and somatic hypermutation (SHM) of the B cell receptor (BCR) take place. The production of high affinity class switched autoantibodies in these ELSs contributes to the local maintenance of the disease process and is often associated with more severe clinical outcomes (1, 2). In fact, we have recently demonstrated that the lympho-myeloid pathotype of RA synovitis, characterized by lymphocyte/myeloid cell infiltration and ELS formation, identified a poor prognosis subgroup of patients with progressive bone erosion and joint destruction. Furthermore, patients with pauci-immune fibroblast-dominant pathotype displayed the poorest response to conventional synthetic disease-modifying antirheumatic drugs in early RA (3, 4).

The unique ability of FDCs to activate B cells in lymphoid tissues stems from their proficiency in regulating the form of antigen and the co-signals B cells encounter during the GC reactions (GCRs). In contrast to conventional dendritic cells that process antigens and present peptides on MHC to T cells, FDCs multimerize monomeric antigens by trapping them in immune complexes (ICs) *via* FDC-Fc-gamma Receptor IIB (-FcγRIIB) and then presenting them polyvalently to BCRs with a characteristic periodic spacing between epitopes of 200–500Å. This allows simultaneous engagement of multiple BCRs which in the presence of the FDC-derived co-signals BAFF, C4b-binding protein, and the complement-derived C3d/CD21 ligand induces T cell-independent B cell activation, GC formation and antigen-specific immunoglobulin secretion (5–8).

FDC differentiation has been mainly studied in the secondary lymphoid tissues (SLT) where a perivascular precursor has been proposed in the late 80s (9, 10) and that was supported later in murine studies favoring a common mesenchymal ancestor of FDCs, marginal reticular cells, fibroblastic reticular cells, and pericytes (11–14). In fact, αSMA^−^ pericytes (type-2 pericytes) exhibit mesenchymal stem cell properties, including multi-lineage potential, away from the vessel wall, in contrast to the αSMA^+^ pericytes associated with the vasculature (type-1 pericytes) (15).

Despite the central role of FDCs in RA ELS formation which correlates with severe clinical manifestations, regulation of FDC development and maturation in the RA synovium and its intersection with the histopathological diversity of RA synovitis are not fully understood. It is intuitively appealing to assume that FDC maturation proceeds through the same developmental cues in SLT and ELS, however, the predestined fate of FDCs in SLT is fundamentally different in nature from that in ELS where the evolving microenvirnment allows plasticity and destination diversion.

Here, we address this, and reveal a reciprocal interplay between PDGF-BB/PDGFR-β and TNF-α/LT-β in driving FDC development/maturation as well as RA synovial fibroid/lymphoid pathotype differentiation, respectively. In addition, we show that pericyte-derived CXCL13 promotes B cell chemotaxis upstream of FDCs, and that activated RA synovial fibroblasts retain ICs and, in an FDC-like manner, induce T cell independent B cell activation and Ig secretion.

## Materials and methods

### RA synovial tissues and histological grading

Synovial tissues were obtained by ultrasound-guided synovial biopsy from DMARD-naïve patients with early (<12 months) RA (*n* = 99), enrolled in the Pathobiology of Early Arthritis Cohort (PEAC) cohort (http://www.peac-mrc.mds.qmul.ac.uk) of the Center for Experimental Medicine and Rheumatology of Queen Mary University of London as previously described (16). All patients fulfilled the 2010 EULAR criteria for RA (17). Patients had clinically defined synovitis with duration of symptoms <12 months and were all naïve to corticosteroids, sDMARDs or biologic therapies. Upon enrollment, patients underwent ultrasound-guided synovial biopsy of a clinically active joint (16). All procedures were performed following written informed consent and were approved by the hospital's ethics committee (REC 05/Q0703/198). Synovitis was semi-quantitatively assessed (0–9) according to a previously validated score (Krenn) (18). Sequentially cut sections were stained with anti CD20cy (L26), CD3 (F7.2.38), CD68 (KP1), and CD138 (MI15) all from DAKO and each slide underwent semi-quantitative scoring (0–4), as previously reported (1). Synovial pathotypes were defined as (i) Lymphoid (L) CD20 ≥ 2 and/or CD138 > 2; (ii) Myeloid (M) CD68SL ≥ 2, CD20 ≤ 1 and/or CD3 ≥ 1, CD138 ≤ 2; and (iii) Pauci-immune/Fibroid (F) CD68SL < 2 and CD3, CD20, CD138 < 1.

### RA synovial fibroblasts propagation and stimulation

Rheumatoid arthritis synovial fibroblasts (RASF) were isolated from synovial tissues obtained from RA patients. After discarding fat and dense fibrous tissues, the synovium was dilacerated with tweezers, and cut into small pieces. The RA synovial pieces were then digested with 1.5 mg/ml Dispase II (Gibco/Invitrogen, Paisley, UK) at 37°C in complete cell culture medium made of Dulbecco's modified Eagle's medium (DMEM) supplemented with 10% fetal calf serum (FCS), 50 IU/ml penicillin-streptomycin, and 10 mM HEPES buffer (Gibco/Invitrogen, Paisley, UK) overnight under rotation. Following enzymatic digestion, the remaining finely minced synovial tissues and detached cell-suspension were passed through a cell strainer and washed with complete DMEM by centrifugation at 400 × *g* for 10 min. The pellet containing synovial cells was collected, re-suspended in complete DMEM and plated into 75-cm^3^ tissue-culture T75 flasks in a 5% CO_2_-humidified incubator at 37°C. The cells were left in culture flask for 7 days including removal of non-adherent cells every other day, and when adherent cells reached 90% confluency, they were passaged at dilution 1/3 into fresh T75 culture flasks by 0.25% trypsin/EDTA (Sigma, Poole, UK) treatment. RASF were used between passages 4 and 8. The RASF were treated with 300 ng/ml PDGF-BB or 100 ng/ml TNF-α + 100 ng/ml LT-αβ for 6 days, then RNA extracted and the differential expression of the FDC related genes was quantified by qPCR using Livak's comparative DD C_T_ method (19).

### Synovial organ culture and IL-6 measurement

RA synovial biopsies were cut into small homogenous pieces (~20–40 mg) avoiding fat and connective tissue. The synovial pieces were gently transferred into cell culture inserts (Millicell, PIHA01250) mounted in 24-well plates containing 600 ul RPMI with 10% FCS. A drop of medium was added on top of the tissues and the plates were incubated for 24 h at 37°C. After 24 h, 300 μl from each well were collected (baseline) and replaced with 300 μl medium supplemented with 300 ng/ml PDGF-BB for further 24 h then culture supernatants were collected. RASF were also stimulated with 300 ng/ml PDGF-BB for 6 days and the culture supernatants were collected. IL-6 levels in the culture supernatants were measured using Human IL-6 Quantikine ELISA Kit.

### Flow sorting of tonsillar single cell suspensions

Tonsillar single cell suspensions were prepared by enzymatic digestion in a mixture of collagenase and DNAse. Briefly, the tonsils were cut into 2 × 2 mm pieces and digested in a cocktail of 2 mL RPMI with 10% FCS + 1.5 mL of 22 mg/mL collagenase D + 0.5 mL of 5 mg/mL DNase I [thus, collagenase D at a final concentration of 8 mg/mL and DNase I at a final concentration of 625 μg/mL] at 37°C for 1 h. The digested tissues were passed through 40 μm strainer (Corning Falcon, Cat#: 352 235) and the cells were spun down at 1,200 rpm for 10 min. The cell pellets were collected and resuspended in cold flow buffer composed of PBS containing 2% (v/v) fetal bovine serum (FBS) and 0.05% sodium azide (NaN_3_). Viability was determined using zombie Aqua (Biolagend, 423101) diluted 1:1,000 in flow buffer then used to stain 1 × 10^6^ cells in 100 μl.

Cells, 1 × 10^6^ cells/100 ul, were fixed in paraformaldehyde-based fixation reagent (ThermFisher, GAS001S5) for 30 min on ice. The cells were then washed in 3 ml flow buffer, and subsequently four single cell suspensions, 5 × 10^6^ each, were stained with one of the following Ab combinations: AF488-NG2/AF647-αSMA; AF488-CNA.42/AF647-NG2; AF488-CNA.42/AF647-αSMA; or AF488-CNA.42/AF647-CD21/CR2. Approximately 1 ug Abs per × 10^6^ cells were used and the cells were stained on ice for 30 min. Finally, the cells were washed with flow buffer and resuspended at a concentration of 1 × 10^6^/ml in flow buffer for sorting on BD FACSAria II and dot blots were generated and analyzed on the FlowJo Cytometry Software. Spectral overlap between the fluorochromes was compensated electronically by single color control samples.

### RNA extraction and qPCR

Total RNA was extracted from RA synovial and tonsillar sorted cell lysates using the RNeasy Mini Kit (Qiagen, 74104) following the manufacturer's instructions. Pelleted cells were first lysed in RLT buffer with 1% β-Mercaptoethanol (Sigma, M3148-25ML) and the lysed samples were homogenized using QIAshredder spin columns. Ethanol 70% (Sigma-Aldrich, 51976-500ML-F) was added to the homogenized lysate and transferred to an RNeasy spin column and centrifuged for 15 s at 10,000 rpm and the flow through the column was discarded. The supplied RW1 buffer (700 μl) was added to the column and spun for 15 s at 10,000 rpm. The column was washed twice with 550 μl RPE buffer, and finally, RNA was eluted in 30–40 μl of RNase-free water. The amount and quality of RNA was determined by measuring the OD260 and OD280 using NanoDrop (Thermo-Fisher, 2000/2000c Spectrometer).

Cloned DNA (cDNA) was generated from the extracted RNA using High-Capacity cDNA Reverse Transcription kit (Applied Biosystems, 4368814). The reaction mix was prepared according to the manual provided with the kit. The Master Mix of the reaction was prepared on ice using: 2.0 μL of 10X RT buffer, 0.8 μL of 25X dNTP mix (100 mM), 2.0 μL of 10X RT random primers, 1.0 μL of MultiScribe™ reverse transcriptase, and 4.2 μL of nuclease-free H_2_O. The total volume of the reaction was adjusted to 20 μl [10 μl of reaction mix plus 10 μl of RNA solution]. cDNA was generated using 96-well GeneAmp 9700 thermal cycler (ThermoFisher) with a cycle setup of 25°C for 10 min, 37°C for 160 min, 85°C for 5 min, and 4°C overnight. cDNA was stored at −20°C until used for qPCR.

Real time qPCR reactions were set up using the gene specific TaqMan probes (Applied Biosystems) listed in Table 1. The total volume of the qPCR reaction was adjusted to 10 μl by adding: 0.5 μl primer probe, 5.0 μl Applied Biosystems Master mix, 1.5 μl cDNA (100 μg) template, and 3.0 μl nuclease free water. The qPCR was carried out in a 7900HT Real-Time PCR System (ThermoFisher, 4329001). The cycle parameters were set up at 2 min hold at 50°C, 95°C for 10 min, 95°C for 15 s, and 60°C for 1 min for 40 cycles. The cycle thresholds (Ct) of the qPCR were automatically calculated using the SDS software 2.3 (Applied Biosystems). The fold changes in treated vs. untreated samples were calculated using Livak's double delta (ΔΔCt) equation [2^−Δ*ΔCt*^] (19, 20); where ΔΔCt = [Ct (gene of interest) —Ct (Housekeeping gene) of the treated sample] – [Ct (gene of interest) —Ct (Housekeeping gene) of the control sample].

**Table 1:** TaqMan gene expression assays used in the study (Thermo-Fisher Scientific Cat Number 4331182).

**Gene symbol**	**Protein**	**Assay ID**
ACTA2	αSMA	Hs00426835_g1
COL1A1	Collagen type 1	Hs00164004_m1
CR1	CD35/CR1	Hs00559348_m1
CR2	CD21L/CR2	Hs00153398_m1
CSPG4	NG2	Hs00361541_g1
CXCL13	CXCL13	Hs00757930_m1
FBXO2	FBXO2	Hs00201792_m1
FCGR2B	CD32/FcγRIIB	Hs01634996_s1
GAPDH	GAPDH [housekeeping protein]	Hs02786624_g1
PDGFRB	PDGFR-β	Hs01019589_m1
THY1	Thy1	Hs00174816_m1
TNFRSF1A	TNF receptor	Hs01042313_m1

### Immunohistochemistry and confocal imaging

Synovial tissues with ELSs from RA patients and tonsils from patients undergoing tonsillectomy were embedded in O.C.T (Sakura) and 10 μm cryo-sections were cut and fixed in ice cold acetone. In addition, cytospin preparation of RA synovial fibroblasts and NG2^+^ pericytes were prepared. The slides were rehydrated and blocked with 2% horse serum (Jackson ImmunoResearch, 008-000-121) in a humidified chamber, incubated at room temperature with primary Abs for 2 h, followed by 3X wash in PBS then incubation with secondary Abs for 1 h. After incubation with the secondary Abs, the slides were washed in PBS 3X, dried and mounted with Vectashield Antifade Mounting Medium (Vector Laboratories), cover-slipped, and examined with a Leica TCS SP2 AOBS confocal laser-scanning microscope. Four lasers (405, 488, 543, and 633 nm) were used and far-red emission was converted into pseudo-color. Parameters were adjusted to scan at 1,024 × 1,024-pixel density and 8-bit pixel depth. Emissions were recorded in separate channels, and digital images were captured and processed with Leica Confocal Software Lite. The Abs used in this study are listed in Table 2 and their concentrations ranged between 5 and 10 μg/ml.

**Table 2:** List of Abs used in this study.

**Primary Abs:**						
**Clone**	**Reactivity**	**Target**	**Host**	**Format**	**Vendor**	**Cat. No**
CNA.42	Human	FDC	Mouse	Unconjugated (IgM)	ThermoFisher Scientific	14-9968-82
7D6	Human	FDC [CD21 Long Isoform]	Mouse	Unconjugated	Dendritics	DDX0120
F95	Human/mouse	Peptidyl citrulline	Mouse	Unconjugated (IgM)	EMD millipore	MABN328
1F8	Human	CD21	Mouse	Unconjugated	Dako-Agilent	M0784
CR2/1952	Human	CD21	Mouse	AF 647	Novus biologicals	NBP260733AF647
L26	Human	CD20	Mouse	Unconjugated	Dako-Agilent	M0755
B-Ly1	Human	CD20	Mouse	RPE	Dako-Agilent	R7013
MECA-79	Human	Peripheral Node Addressin (PNAd)	Rat	AF 594	Biolegend	120805
9.2.27	Human	Neural/Glial Antigen 2	Mouse	Unconjugated	Invitrogen	14-6504-82
1A4	Human	Actin, α-Smooth Muscle	Mouse	Cy3	Sigma-Aldrich	C6198
1A4	Human	Actin, α-smooth muscle	Mouse	AF 488	eBioscience	53-9760-82
AS02	Human	Fibroblast Antigen [Thy1/CD90]	Mouse	Unconjugated	Sigma-Aldrich	CP28-200UG
Polyclonal	Human	CD45	Rabbit	Unconjugated	abcam	ab10558
Polyclonal	Human	CD31	Rabbit	Unconjugated	abcam	ab28364
Y92	Human	PDGFR-β	Rabbit	Unconjugated	abcam	ab32570
Polyclonal	Human	CXCL13/BLC/BCA-1	Goat	Unconjugated	R&D Systems	AF801
Polyclonal	Mouse/human	PAD4	Rabbit	Un-conjugated	Abcam	ab50247
**Secondary Abs and other stains:**						
**Clone**	**Reactivity**	**Target**	**Host**	**Format**	**Vendor**	**Cat. No**
Polyclonal	Mouse	IgM μ chain	Donkey	AF 594	Jackson ImmunoResearch	715-585-140
Polyclonal	Mouse	IgM μ chain	Donkey	AF 488	Jackson ImmunoResearch	715-545-140
Polyclonal	Mouse	IgM μ chain	Donkey	AF 647	Jackson ImmunoResearch	715-605-020
Polyclonal	Mouse	IgG (H + L)	Donkey	F (ab')2—AF 488	Jackson ImmunoResearch	715-546-150
Polyclonal	Mouse	IgG (H + L)	Donkey	F (ab')2—AF 594	Jackson ImmunoResearch	715-585-150
Polyclonal	Mouse	IgG (H + L)	Donkey	F (ab')2 - AF 647	Jackson ImmunoResearch	715-606-150
Polyclonal	Rabbit	IgG (H + L)	Donkey	F (ab')2—AF 647	Jackson ImmunoResearch	711-606-152
Polyclonal	Rabbit	IgG (H + L)	Donkey	F (ab')2—AF 594	Jackson ImmunoResearch	711-586-152
Polyclonal	Rabbit	IgG (H + L)	Donkey	F (ab')2—AF 488	Jackson ImmunoResearch	711-545-152
Polyclonal	Goat	IgG (H + L)	Donkey	F (ab')2—AF 488	Jackson ImmunoResearch	705-546-147
N/A		Biotin		Sreptavidin AF 488	ThermoFisher Scientific	S11223
N/A		Double Stranded DNA		DAPI	Merck	10236276001

### Immunocytochemistry

Approximately 1 × 10^4^ flow sorted NG2^+^/αSMA^+^ tonsillar pericytes were cultured in 8 well Nunc™ Lab-Tek™ II Chamber Slide™ System (154534PK). The cells were treated with 300 ng/ml PDGF-BB for 72 h then washed and labelled with 10 μg/ml CNA.42 mAb for 1 h at room temperature. After washing of the primary antibody, AF594 anti-mouse IgM or AF594 mouse IgM isotype control at 10 μg/ml and DAPI for nuclear staining were added for 30 min. Similarly, Thy-1 and αSMA expression in RA synovial fibroblasts (RASF) were confirmed by immunocytochemistry prior to treatment with PDGF-BB, TNF-α, and LT-β and qPCR analysis. RASF (~1 × 10^4^ cell/well) were cultured in slide chambers and labelled with anti-Thy-1 Ab. The cells were fixed and permeabilized with fix and perm kit (Thermofisher, GAS003), then stained with anti-mouse IgG AF488, DAPI and anti-αSMA-Cy3 Ab for 30 min. The slides were washed, mounted and cover slipped then examined by confocal microscopy.

After image acquisition, ImageJ (US National Institutes of Health, https://imagej.nih.gov/ij/) was used to analyse CNA.42 expression on PDGF-BB treated pericytes and control conditions. Briefly, the color channels in the RGB files were split using the “color split channels” command then the threshold of the red channels (CNA.42) was adjusted using the “image, adjust threshold” command. The mean fluorescence intensity (MFI) and the percentage area of pixels above threshold were then calculated using the “analyse, measure” command. MFIs multiplied by the percentage areas were calculated for the different conditions and represented in a histogram.

### Transmission electron microscopy (TEM)

The ultrastructure of TNF-α/LT-β activated RA synovial fibroblasts loaded with HRP ICs and ultrathin sections of the RA synovium were investigated using TEM. RA synovial fibroblasts (RASF) were incubated with HRP-rabbit anti-HRP ICs for 2 h on ice. The ICs consisted of 100 ng HRP (Sigma-Aldrich, P8375) and 600 ng AffiniPure Rabbit Anti-Horseradish Peroxidase (Jackson ImmunoResearch, 323-005-021). The cells were then washed three times then incubated with Peroxidase AffiniPure Goat Anti-Rabbit IgG (Jackson ImmunoResearch, 11-035-144) to amplify the HRP signal in a specific manner. Diaminobenzidine (DAB) (Vector Labs, SK-4100) was used to develop the HRP from both rounds of labeling. HRP-loaded RA synovial fibroblast pellets and ~1 mm^3^ RA synovial biopsies were fixed with 1% paraformaldehyde + 0.9% glutaraldehyde in 0.1 M cacodylate buffer, post-fixed in 1% OsO_4_ in 0.1 M cacodylate buffer for 90 min, dehydrated in a graded series of ethanol, infiltrated, and embedded in PolyBed 812 epoxy resin. Approximately 800 Å thick sections from the embedded synovial tissues and pelleted cells were mounted on slotted grids and stained with lead citrate and uranyl acetate for morphological evaluations or detection of labeling of the RASF with HRP ICs. The sections were studied with a JOEL electron microscope at 50–100 kV.

### Trans-well migration assay

The impact of PDGF-BB and TNF-α/LT-β on RA synovial stromal cell migration was assessed using a trans-well migration assay. Twenty-four trans well plate with 6.5 mm diameter inserts fitted with 5 μm pore polycarbonate membrane filters (Corning Costar, Cambridge, MA, USA, 3421) were used. The synovial stromal cells (~1 × 10^6^), in triplicates per condition, were added to the upper well in DMEM F12 and left untreated, or treated with 300 ng/ml PDGF-BB alone or in addition to 100 ng/ml TNF-α + 100 ng/ml LT-αβ. Following overnight incubation at 37°C, the trans-well insets were removed from the plates and the cells in the filters fixed with 70% ethanol. The trans-wells were left to dry then stained with Giemsa stain for 30 min. The filters were carefully washed with distilled water, left to dry and the migratory cells in 6 fields were counted under the microscope.

### *In vitro* germinal center reactions

Naive “untouched” human B cells were stimulated with anti IgM in the form of ICs as we previously described for the murine B cells using anti IgD ICs (5). The cells were purified by negative selection on LS MACS columns using the Naive B Cell Isolation Kit II (130-091-150; Miltenyi Biotec). TNF-α/LT-β-stimulated RA synovial stromal cells (RASF) were loaded with 100 ng/ml goat anti-human IgM (Bethyl Laboratories, A80-100A) in the form of ICs with rabbit anti-goat IgG, Fc fragment specific (min X Hu Sr Prot) (Jackson ImmunoResearch, 305-005-046) at a ratio of 1:4. Anti IgM IC-loaded RASF were used to stimulate 10^6^ purified B cells in 1 ml of RPMI at a ratio of 1 RASF to 2 B cells. Controls included unstimulated B cells and B cells stimulated with 100 ng soluble anti IgM. Culture supernatant fluids were collected after 6 days and the human IgM levels were measured using Human IgM ELISA Kit (Bethyl Laboratories, E88-100). Control wells containing anti IgM without cells were included and backgrounds registered in the ELISA were subtracted.

### CNA.42 specificity determination using HuProt™ human proteome microarrays

CNA.42 specificity was assessed in Cambridge Protein Arrays Ltd., Babraham Research Campus, Cambridge, UK using human proteome arrays (HuProt Ver2). The HuProt™ human proteome microarray contained around 20,000 unique, newly re-sequenced and individually purified recombinant human proteins representing 87% of the human proteome. The recombinant proteins are expressed in yeast (*S. cerevisiae*), then the N-terminal GST- and His6-tagged proteins are purified and printed on glass slides in duplicate, along with control proteins (GST, BSA, Histones, IgG). HuProt arrays for CNA.42 [HuProtTM barcode 1300017931], positive control, anti-Glutathione S-transferases (anti-GST) [HuProtTM barcode 1300017932] and negative control with no sample were blocked in 2% BSA/PBS-Tween 0.1%, then CNA.42 at a concentration of 10 μg/ml and biotinylated anti-GST antibody at a concentration of 0.5 μg/ml were added to the arrays and incubated at room temperature for 2 h with gentle shaking. The arrays were rinsed 3X with 5.0 ml of PBS-T (PBS, 0.1% Tween-20), then Cy3-conjugated AffiniPure goat anti-mouse IgM μ chain reagent (final concentration 3 μg/ml in blocking buffer) and Streptavidin-647 (final concentration 1 μg/ml in blocking buffer) were incubated for 2 h at room temperature on the CNA.42 and GST HuProt™ arrays, respectively. The HuProt™ arrays were washed with PBS-T 3X for 10 min with gentle shaking, and the slides were rinsed with ddH_2_O to remove residual PBS-T. The slides were dried by spin-down, and scanned at 543 nm (detection of interactions from CNA.42) and 633 nm (detection of GST staining of all proteins) with a resolution of 10 μm and fluorescence data collected. Genepix Pro 4 software was used to align the human proteins spotted with the array positioning file. After aligning each of the 48 subarrays using fluorescently conjugated control spots, the spots were repositioned and resized by the software to yield the best fit. Fluorescence intensities for each spot on the array were determined by the software and saved. After subtraction of the negative control array data, the Hit Score (HS), the interaction score (IS), and the signal to noise (S/N) ratio were calculated. The HS measures the signal strength of CNA.42 relative to other human protein spots which act as a general negative control. The IS measures the signal strength obtained from CNA.42 binding to its target protein relative to the amount of immobilized protein, as judged by the staining intensity for the corresponding GST tag.

### *In situ* hybridization

*In situ* hybridization of the FBXO2 RNA in tonsillar tissues was carried out using The RNAscope^®^ Multiplex Fluorescent Reagent Kit 2.0 (Cat. No. 323100; Advanced Cell Diagnostics ACD) (21). Alexa Fluor 488-conjugated FBXO2 probe was designed and synthesized by ACD [17ZZ, Hs-FBXO2 targeting 464-1699 of The FBXO2 mRNA; NCBI Ref Seq: NM_012168.5]. negative and positive control probes were included. Cryosections of human tonsils (~6 um thick) were fixed in fresh prechilled 4% paraformaldehyde for 30 min then rinsed twice in PBS. The slides were dehydrated using 50, 70, and 100% ethanol 5 min each, then left to air-dry. The slides were covered with 5 drops of protease IV to promote permeabilization and increase target accessibility; then, the target, positive, and negative probes were added, and RNA/probe hybridization was maintained for 2 h at 40°C in a hybridization oven. Signal preamplifiers, and amplifiers were then added for 15–30 min at 40°C then the slides washed twice with PBS. FDCs were labeled in the tissues with 10 μg/ml mouse anti human CD21 (clone IF8) followed by Alexa Flour 594 conjugated F(ab')2 donkey anti mouse IgG. At least three slides of the FBXO2 target, positive control, and negative control were prepared and examined by confocal microscopy.

### Immunoprecipitation of the CNA.42-binding protein

Cell membrane proteins of tonsillar single cell suspension were extracted according to the Membrane Protein Extraction Kit (Thermo Fisher Scientific) protocol. The membrane proteins were concentrated using 10 kDa Amicon Ultra centrifugal filters (Millipore), and quantified using the BCA protein assay as per manufacturer's recommendations (Thermo Fisher Scientific). Next, 1 mg/ml of the cell membrane extracts were incubated with 70–100 μl Protein L (Thermo Fisher Scientific) beads at 4°C for 1 h. The beads were subsequently removed following 3X cycles of washing in PBS, resulting in pre-clear samples free from non-specific bead-bound proteins. Similarly, 15 μg of the CNA.42 antibody was incubated in 70-100 μl of Protein L at room temperature for 2 h. Following 3X washes in PBS, the CNA.42 bound Protein L beads were incubated overnight with the pre-clear samples for immunoprecipitation. Immunoprecipitated proteins were washed in PBS and assessed by western blotting. In addition, the immunoprecipitated bands at 120 kD were excised and sent for mass spectrometry.

### Inhibition of FBXO2 expression by small interfering RNA (siRNA)

Triplicates of CNA.42 expressing cell line CEM/C2 (ATCC^®^ CRL-2264™) (22) were plated in a 24-well plate (1 × 10^6^ cells/well) and were used at 75% confluence at the time of transfection. The cells were treated with 1 uM Accell Human FBXO2 siRNA (Dharmacon), 1 μM Accell Non-Targeting Control Pool (Dharmacon) or left in RPMI or Accell media only. After incubation at 37°C for 72 h, cell lysates were prepared using RIPA Buffer (Sigma) and quantitated by Pierce™ BCA Protein Assay Kit (Thermofisher Scientific).

### Western blotting

Forty to 60 μg of tonsillar tissue lysates, CNA.42-immunoprecipitated proteins, or CEM/C2 (ATCC^®^ CRL-2264^™)^ cell lysates were separated by SDS-polyacrylamide gels (90 V for 1 h) then transferred (100 V for 1 h) to nitrocellulose membranes. The membranes were probed overnight at 4°C with 2 μg/mL of CNA.42 or 1:200 FBXO2 rabbit polyclonal antibody (Proteintech). After washing, the membranes were incubated for 1 h at room temperature with HRP-goat anti-mouse IgM (1:2,000) or peroxidase conjugated goat anti-rabbit IgG (1:2,000) (Jackson Immunoresearch) and anti-GAPDH (1:10,000) to detect CNA.42, FBXO2, and GAPDH, respectively. Proteins bound to the antibodies were then visualized using chemiluminescent substrate (ECL; Bio-Rad).

### Densitometry of the western blots

FBXO2 and CNA.42 abundance in the western blots of the siRNA transfected CEM/C2 (ATCC^®^ CRL-2264™) cells and controls was compared using ImageJ. First, the images were converted into 8-bit grayscale format, then the lanes were selected and plotted using the “analyse, gels, plot lanes” command. The hight of the generated peaks represented the intensity of the bands and the base represented the extent of the bands. The peaks of the target and housekeeping proteins were both measured and the ratio of the target to the housekeeping was calculated and presented in histograms.

### RNA-Seq analysis

Expression of the FDC-related genes in the rheumatoid synovium was analyzed using RNA-Seq data generated from RA synovial tissue and matched blood samples enrolled in the PEAC cohort https://peac.hpc.qmul.ac.uk/ (3, 4). Briefly, transcript abundance was derived from FASTQ files over GENCODE v24/GRCh38 transcripts using Kallisto v0.43.0. Transcript abundances and average transcript lengths were imported into R using Bioconductor package tximport 1.4.0 and summarized over NCBI RefSeq transcript isoforms. Imported abundances were normalized in R, including a correction for average transcript length and incorporating batch, sex, and pathotype as model covariates, using DESeq2 1.14.1. Transcript abundances underwent regularized log expression (RLE) transformation and depicted on the Y axis whereas the synovial pathotypes were shown on the X axis. Differential expression analysis based on negative binomial distribution using regression models of normalized count data was performed using DESeq2 and a likelihood ratio test to compare variation between pathotype groups in the synovium followed by pairwise comparisons between Lymphoid, Myeloid and Fibroid groups. *p*-values were converted to Q values based on Benjamini–Hochberg false discovery rate (FDR), using FDR cut-off set at Q < 0.05 to define differentially expressed genes.

Hierarchical clustering of the FDC module genes was performed using Euclidean distance metric and Ward's linkage method and plotted using the ComplexHeatmap package 2.12.1 in R. Color track annotations for histology data for CD3, CD20, CD68L/SL (lining/sublining), CD138 and overall pathotype were included to aid interpretation.

### Statistical analysis

Statistical analysis was performed using GraphPad Prism7.03. Each experiment was performed at least in triplicate. Data were presented as means ± standard deviations (SD) or standard error of means (SEM) as specified in the results. The significance of difference between two means (*p*-value) was calculated using the student *T*-test and a *p*-value of <0.05 was accepted as significant.

### Supplemental material

Supplementary Figure S1 shows colocalization of the FDC differentiation and maturation markers in the tonsil.

## Results

### RA synovial FDCs display pericyte and fibroblast markers in the perivascular space but not in established follicles

**Figure 1:**
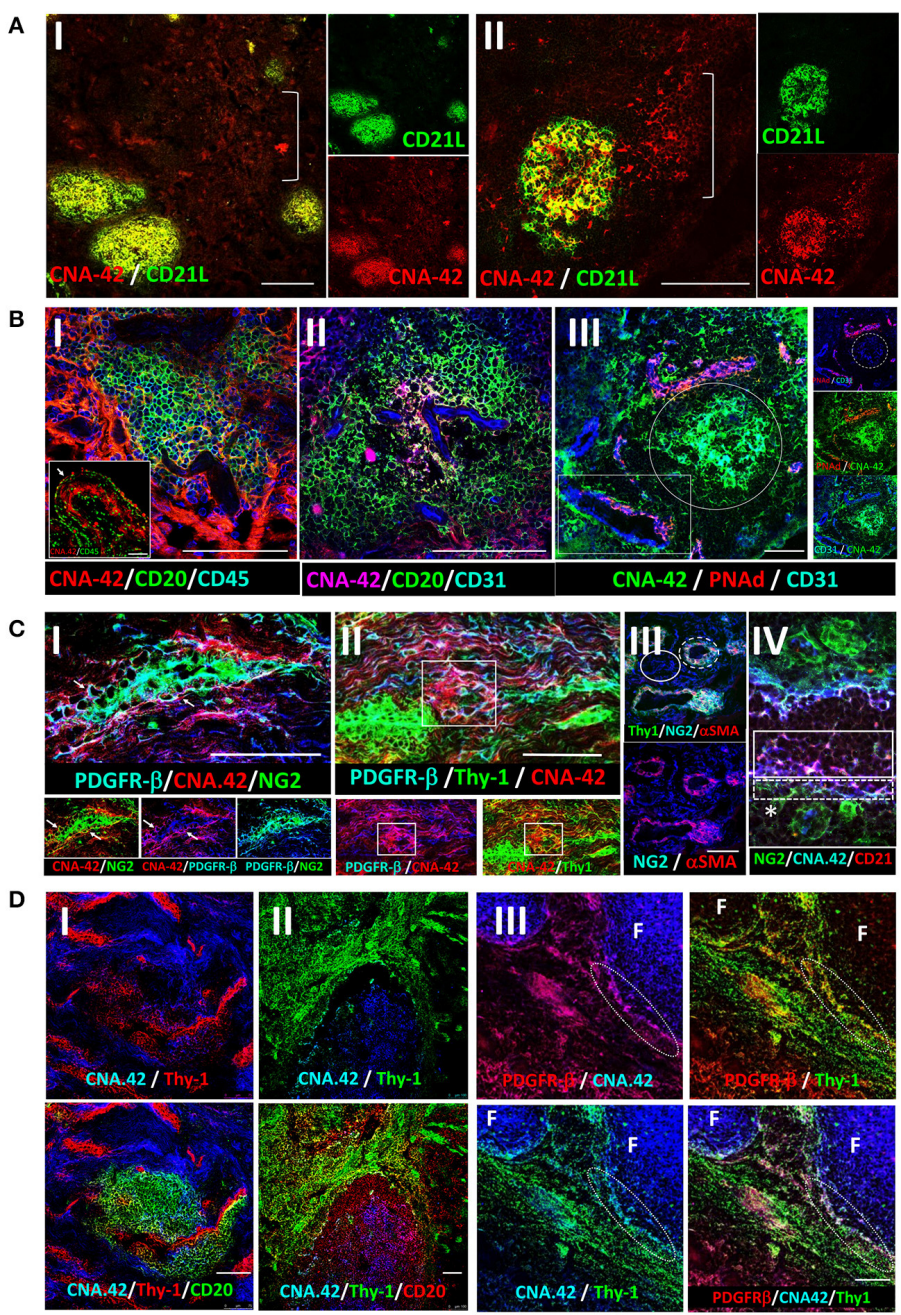
Immunohistochemical localization of human synovial and tonsillar FDCs in the perivascular space vs. established follicles. **(A)** CNA.42 distribution in the tonsil **(A-I)** and RA synovium **(A-II)**. Labeling of human FDCs with the mAb CNA.42 (red) colocalizes with an Ab specific for the FDC-restricted CD21 long isoform (CD21L, green) in FDC reticula. The white bracket borders extra-reticular FDCs that are CNA.42^+^/CD21L^−^. **(B)** RA synovial CNA.42^+^ cells (red) are: **(B-I)** associated with CD20^+^ B cells (green) and do not colocalize with CD45 (blue). The inset in the lower left corner shows the synovial intima with CNA.42^+^ fibroblasts (red/white arrow) arranged side by side with CD45^+^ intimal macrophages (green) in the lining layer. **(B-II)** directly related to the CD31^+^ BVs (blue) within CD20^+^ B cell follicles (green), and **(B-III)** directly located in juxtaposition with the CD31^+^ (blue)/PNAd^+^ (red) HEVs (white rectangle). The white circle borders established CNA.42^+^ FDC reticulum (green) detached/distant from the vascular walls. **(C)** Early FDC markers in the RA synovial perivascular space: **(C-I)** Synovial labeling with the FDC marker CNA.42 together with the pericyte markers NG2, αSMA, and PDGFR-β shows that CNA.42 is expressed by cells immediately adjacent to the pericyte layer of the BVs (white arrows). **(C-II)** The CNA.42^+^/PDGFR-β^+^ early FDCs also express the fibroblasts marker Thy-1 (white rectangle) which is similarly expressed on the BV-associated type-1 pericytes (dashed while circle) but not on the dissociated type-2 pericytes (complete white circle) **(C-III)**. **(C-IV)** Pericyte-derived differentiating FDCs progressively acquire CNA.42 and CD21/CR2 expression. NG2^+^ pericytes (green) are marked by a star (*); and differentiating NG2^+^ (green)/CNA.42^+^ (blue) and CNA.42^+^ (blue)/CD21^+^ (red) FDCs are indicated by dashed and complete white boxes, respectively. **(D)** Co-labeling of the FDC marker CNA.42 and the fibroblast marker Thy-1 in established follicles (mature FDCs) of RA synovial ELS **(D-I)** and tonsils **(D-II)**. The fibroblast marker is significantly lost in mature FDCs populating established CD20^+^ B cell follicles. **(D-III)** Co-localization of CNA.42 (Blue), Thy-1 (green), and PDGFR-β (red) in the follicular (F) and inter-follicular areas. The dotted oval demarcates the loss of PDGFR-β and Thy-1 expression at the follicular border. Single channel, dual and triple overlays are shown in the different panels. Scale bare = 100 um.

The mAb CNA.42 uniquely recognizes immature and mature human FDCs in SLTs, cell lines, and histopathological specimens (22–28). To elucidate the tissue distribution and phenotypic profile of the RA synovial FDCs, we labeled sections from ELS^+^ RA synovial tissues with CNA.42 and compared its localization/colocalization in the RA synovium with SLTs (tonsils). As illustrated in Figure 1A-I (tonsil) and Figure 1A-II (RA synovium), CNA.42 colocalizes with an Ab specific for the FDC-restricted CD21 long isoform (CD21L) in the FDC reticula; and additionally, labels extra-reticular FDCs that are CNA.42^+^/CD21L^−^. Furthermore, CNA.42 in the RA synovium (Figure 1B) displayed features typical of FDCs in SLTs where they localize with CD20^+^ B cells and lack the expression of CD45 (29). Interestingly, CNA.42 labeled synovial intimal fibroblasts but not macrophages that were CD45^+^ (Figures 1B-I,II). In addition, CNA.42 was directly related to the CD31^+^ synovial vasculature including the PNAd^+^ high endothelial venules (HEV) (Figure 1B-III) suggestive of a perivascular origin of synovial FDCs. In fact, remnants of degenerate blood vessels (CD31^+^) within CNA.42^+^ dendritic reticula can be seen in the perivascular space (Figure 1B-III).

The juxtaposition of CNA.42 to the synovial vasculature prompted our reasoning that PDGFR-β^+^ pericytes could represent the early FDC precursors in the RA synovium. To test this, we labeled the RA synovium with CNA.42, and the pericyte markers NG2, αSMA, and PDGFR-β and compared their distribution in the perivascular space. As shown in Figure 1C, CNA.42 was expressed by cells immediately adjacent to the mural layer of the blood vessels (BVs) where the pericyte markers NG2 and PDGFR-β were co-expressed with the FDC CNA.42 at this stage (Figure 1C-I). Moreover, the CNA-42^+^/PDGFR-β^+^ early FDCs expressed the fibroblast marker Thy-1 (Figure 1C-II) which together with αSMA colocalized with the vessel wall associated type-1 pericytes (NG2^+^/αSMA^+^) but not with the dissociated type-2 pericytes (NG2^+^/αSMA^−^) (Figure 1C-III). Furthermore, expression of the complement receptor 2 (CD21) was acquired early on the differentiating FDCs, and a transition from NG2^+^/CNA.42^+^ to CD21^+^/CNA.42^+^ FDCs was observable in the perivascular space (Figure 1C-IV).

Critically important, the early association of the FDC CNA.42 and the fibroblast Thy-1 markers was lost in mature FDC reticula of RA synovial ELS (Figure 1D-I). Indeed, this lack of Thy-1 expression on mature FDCs within synovial ELS recapitulates the status in tonsillar SLTs where FDCs and fibroblasts are topographically segregated (Figure 1D-II). Moreover, the lack of Thy-1 staining is associated with loss of PDGFR-β expression in the mature follicles as demonstrated by the sharp demarcation at the follicular border (Figure 1D-III).

Overall, early synovial FDCs express the pericyte and fibroblast markers PDGFR-β, NG2, and Thy-1; whereas, in established follicles, mature FDCs are distinct from fibroblasts and do not express Thy-1 or PDGFR-β.

### PDGF-BB/PDGFR-β and TNF-α-LT-β/TNF-αR-LT-β-R differentially correlate with FDC-related genes expression and synovial pathotypes in RA

The contrast in the colocalization of PDGFR-β with early FDCs in the perivascular space but not with mature FDCs in the lymphoid follicles of the RA synovium triggered our search for mechanistic correlations. Several lines of evidence in murine SLTs indicated that while PDGF-BB/PDGFR-β support early FDC development (13), full maturation of FDCs requires TNF-α and LT-β (30–32). Consequently, we argued that PDGF-BB/PDGFR-β and TNF-α-LT-β/TNF-αR-LT-β-R correlate with distinct sets of genes associated with early and late RA synovial FDC development, respectively.

**Figure 2:**
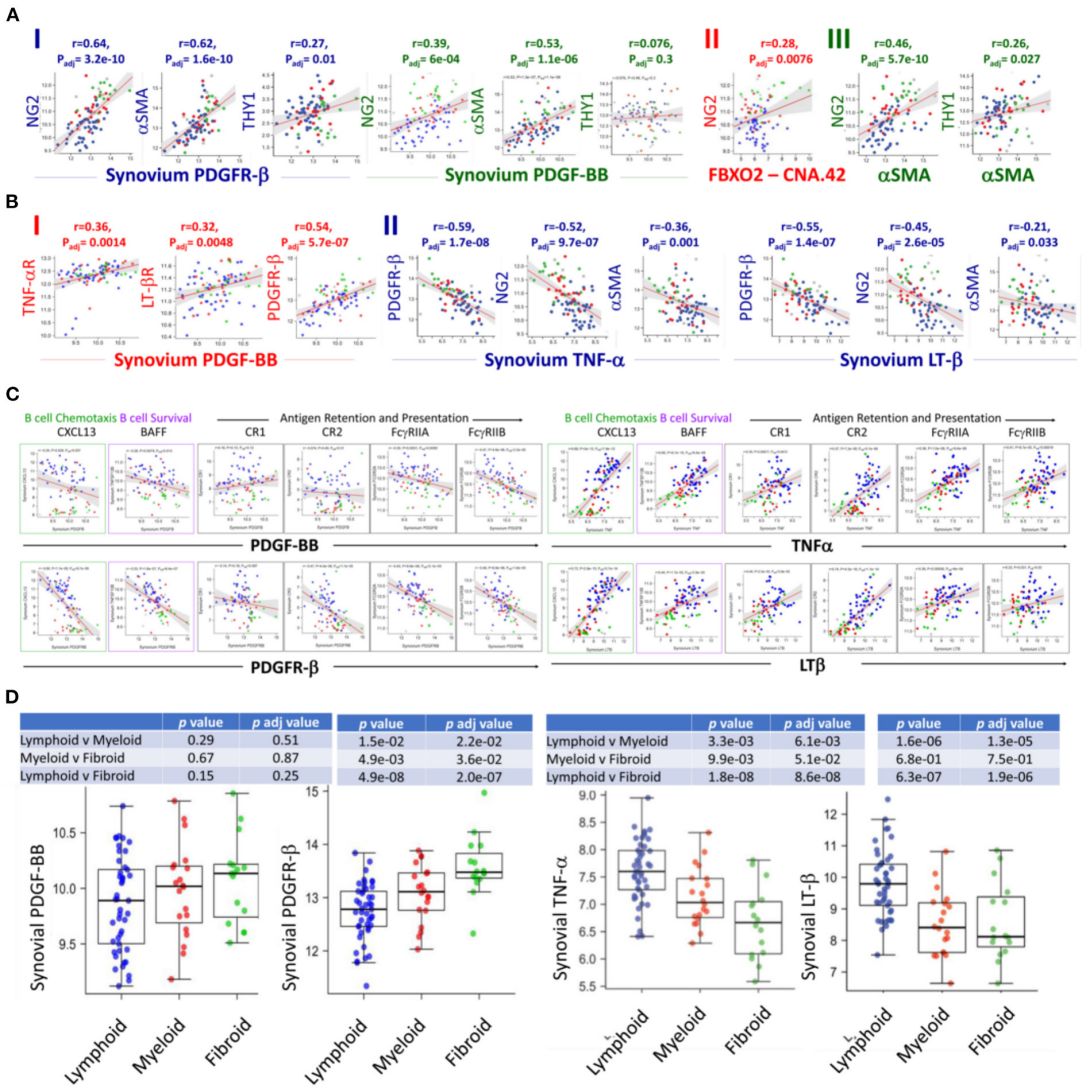
RNA-Seq analysis of the PEAC cohort illustrates the differential correlation of the FDC genes associated with early perivascular and late mature developmental stages in the RA synovium. Strong positive correlations of the pericyte/fibroblast markers NG2, THY1 and αSMA with the PDGFR-β/PDGF-BB axis **(A-I)**, NG2 and FDC-CNA.42/FBXO2 **(A-II)**, and NG2, THY1 and αSMA **(A-III)** in RA. **(B)** Correlations of PDGF-BB and TNFα/LTβ with the expression of each other's receptors and early FDC developmental genes. PDGF-BB positively correlates with its receptor and the TNFα/LTβ receptors **(B-I)**, TNFα and LTβ negatively correlate with PDGFR-β expression and the early FDC markers NG2 and αSMA **(B-II)**. **(C)** Converse correlations of the PDGF-BB/PDGFR-β and the TNF-α/LT-β axes with the expression of mature FDC markers. PDGF-BB/PDGFR-β and TNF-α/LT-β differently correlate with the mature FDC related genes CXCL13 (B cell chemoattractant), BAFF (B cell survival factor), and antigen display and presentation to B cells namely complement receptors (CR1/CD35, CR2/CD21), and Fcg receptors (FcγRIIA/CD32A, FcγRIIB/CD32A). **(D)** Correlation of the RA synovial pathotypes with the expression of PDGF-BB, PDGFR-β, TNF-α, and LT-β. Person correlation coefficient (r) and adjusted *p*-values are shown with the corresponding plots and tables.

Analysis of the RNA sequences generated from the Pathobiology of Early Arthritis Cohort (PEAC) study http://www.peac-mrc.mds.qmul.ac.uk/ indicated that PDGFR-β and PDGF-BB positively correlate with the pericyte/activated fibroblast genes NG2/CSPG4, αSMA/ACTA2, and Thy-1/THY1. In addition, the NG2/CSPG4 significantly correlates with the FDC CNA.42/FBXO2 and αSMA/ACTA2 characteristic of type-1 pericytes (Figure 2A).

The switch from an early pericyte/myofibroblast-related pre-FDCs to fully mature FDCs requires TNF-αR and LTβ-R expression. Remarkably, PDGF-BB positively correlated with the expression of both receptors as well as its own receptor PDFR-β (Figure 2B-I). On the contrary, TNF-α and LT-β negatively correlated with the expression of PDFR-β as well as genes of type 1 pericytes NG2/CSPG4 and αSMA/ACTA2 (Figure 2B-II). Furthermore, PDGF-BB/PDGFR-β and TNF-α-LT-β/TNF-αR-LT-βR inversely correlated with the expression of mature FDC genes associated with B cell chemotaxis (CXCL13), B cell survival (BAFF), and antigen retention and presentation (CR1/CD35, CR2/CD21, FcγRIIA, and FcγRIIB) (Figure 2C). This inverse correlation was further illustrated in their converse association with the fibroid and lymphoid RA synovitis, respectively (Figure 2D).

Altogether, PDGF-BB/PDGFR-β and TNF-α/LT-β correlate with genes associated with early FDCs/fibroid and mature FDCs/lymphoid RA synovial pathotypes, respectively. In addition, PDGF-BB positively correlates with TNF-αR/LT-βR expression, whereas TNF-α/LT-β negatively correlates with the expression of PDGFR-β.

### PDGF-BB and TNF-α/LT-β inversely regulate the expression of early and late FDC developmental genes in synovial stromal cells

**Figure 3:**
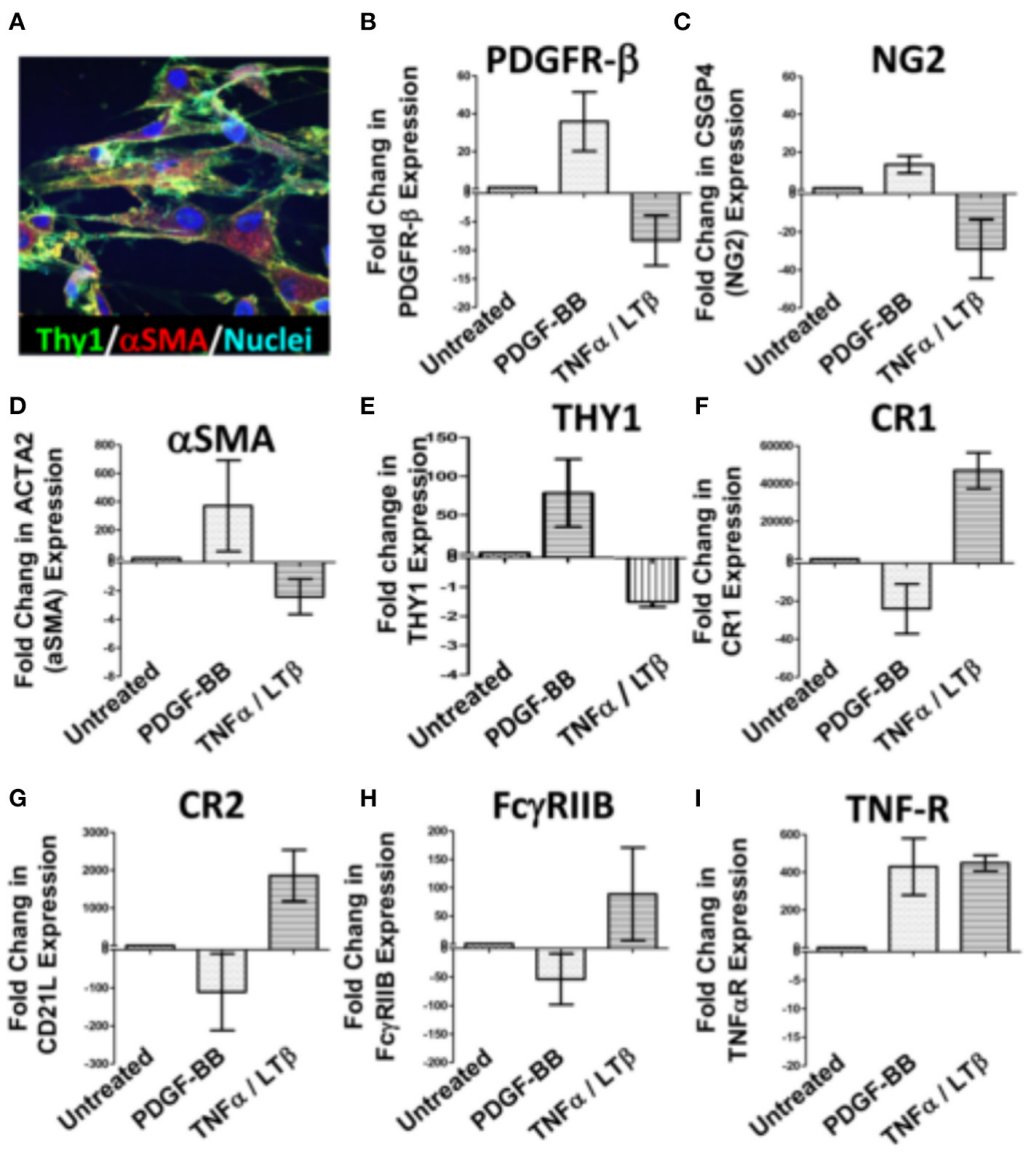
Regulation of synovial stromal cell gene expression by PDGF-BB and TNFα/LTβ. RA synovial stromal cells expressing Thy-1 on the cell membrane and displaying intracellular αSMA labeling **(A)** were treated with 300 ng/ml PDGF-BB or 100 ng/ml TNF-α + 100 ng/ml LT-αβ and the fold change in gene expression compared to untreated cells was calculated by Livak's DD equation. **(B–I)** Show the fold change in PDGFR-β, CSGP4 (NG2), ACTA2 (αSMA), TNFRSF1A (TNF-R), THY1, CR1 (CD35), CR2 (CD21L), FcγRIIB (CD32), and TNF-αR, respectively. Error bars represent the SEM of three different cell cultures.

The divergent correlation of PDGF-BB/PDGFR-β and TNF-α/LT-β with FDC developmental markers and RA synovial pathotypes demonstrated by the RNA-Seq analysis prompted the question “Can these correlations be recapitulated *in vitro*?” To answer this question, we stimulated RA synovial stromal cells (Figure 3A) with PDGF-BB or TNF-α/LT-αβ for 6 days then quantified the differential expression of FDC differentiation genes. Our results demonstrated that PDGF-BB and TNF-α/LT-αβ inversely regulated early [PDGFR-β, αSMA, NG2, THY-1] and late [CR1, CR2, FcγRIIB] FDC-related genes where the early genes were upregulated by PDGF-BB and downregulated by TNF-α/LT-β (Figures 3B–E), whereas, the late maturation genes were induced by TNF-α/LT-β and inhibited by PDGF-BB (Figures 3F–H). Exceptionally, the TNF-αR gene expression was mutually inducible by both PDGF-BB and TNF-α/LT-β (Figure 3I).

On the whole, *in vitro* stimulation of synovial stromal cells with PDGF-BB or TNF-α/LT-β resulted in FDC gene regulation comparable to the correlations deduced from the RNA-Seq analysis of the RA synovial tissues derived from the PEAC cohort.

### Sorted stromal cell subsets distinctively upregulate FDC differentiation and maturation genes upon treatment with PDGF-BB and TNF-α/LT-β, respectively

To this point, data generated from immunolabeling of the RA synovial tissues, bulk RNA-Seq, and *in vitro* stimulation of unsorted synovial stromal cells support a reciprocal role of PDGF-BB and TNF-α/LT-β in FDC differentiation and maturation in the RA synovium. However, these data are short of stromal cell subset specificity, thus, we sought to compare the differential impact of PDGF-BB and TNF-α/LT-β on sorted stromal cell subsets in order to verify induction of individual FDC maturation genes in specific stromal cell subsets.

Sorting enough numbers of RA synovial stromal cells from patients with ELS that allow *in vitro* cultures with several stimulation conditions, replicates and repeats is challenging. Accordingly, we considered flow sorting of the relevant stromal cell populations from tonsillar single cell suspensions gating on the CD45^−^ cells.

Before sorting, we ensured that the distribution of FDC developmental markers in the tonsil is comparable to the RA synovium. Our results (Supplementary Figure S1) indicated that PDGFR-β colocalizes with NG2 around CD31^+^ blood vessels. In addition, the tonsillar tissue displayed both types of pericytes; the NG2^+^/αSMA^+^ type-1 and the NG2^+^/αSMA^−^ type-2. Moreover, PDGFR-β, NG2 and αSMA colocalized with CNA.42 outside the established follicles thus marking the early FDC phenotype.

**Figure 4:**
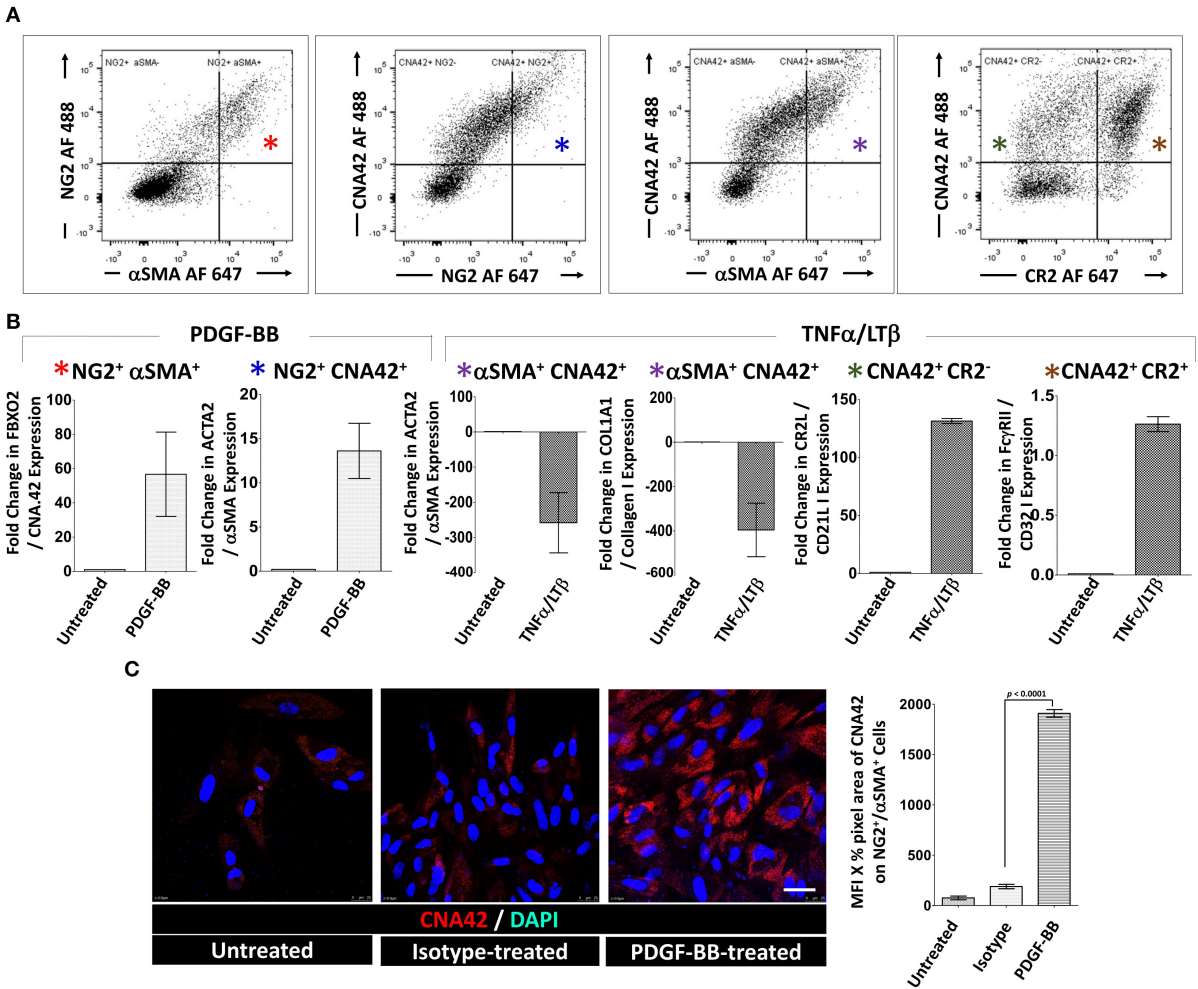
Activation of sorted tonsillar stromal cell subsets with PDGF-BB and TNF-α/LT-β induces early and mature FDC markers *in vitro*. **(A)** The CD45^−^ tonsillar stromal subsets were sorted using combinations of NG2/αSMA, NG2/CNA.42, CNA.42/αSMA and CNA.42/CR2 Abs. **(B)** Type-1 Pericytes [NG2^+^/αSMA^+^; indicated by red * in **(A,B)**], early FDCs; (CNA.42^+^/NG2^+^, CNA.42^+^/αSMA^+^, CNA.42^+^/CR2^−^; indicated in A and B by blue, magenta, and green *, respectively) and mature FDCs [CNA.42^+^/CR2^+^, indicated by brown * in **(A,B)**] were treated with 300 ng/ml PDGF-BB or 100 ng/ml TNF-α+ 100 ng/ml LT-αβ and the fold change in FBXO2 (CNA.42), αSMA, Collagen 1, CR2, and FcγRIIB gene expression compared to untreated cells was calculated by Livak's DD equation and shown in bar graphs. **(C)** Treatment of the NG2^+^/αSMA^+^ type-1 pericyte subset with PDGF-BB in slide cultures for 6 days induced the expression of the FDC marker CNA.42 compared to untreated cells as demonstrated by Immunocytochemistry. Image J quantification of the mean fluorescence intensity (MFI) of CNA-42 of the different conditions is shown in the histogram. Data is representative of three different experiments and is expressed as the mean ± SEM.

Next, we sorted (1) NG2^+^/αSMA^+^ type-1 pericytes (FDC progenitors), (2) CNA.42^+^/NG2^+^, CNA.42^+^/αSMA^+^, and CNA.42^+^/CR2^−^ (early FDCs) and (3) CNA.42^+^/CR2^+^ (mature FDCs) (Figure 4A). The sorted cells were then treated with PDGF-BB or TNF-α/LT-β for 6 days then the relative expression of selected FDC differentiation genes was compared with untreated cells using qPCR. As illustrated in Figure 4B, expression of the early FDC differentiation marker CNA.42 (FBXO2) in NG2^+^/αSMA^+^ type-1 pericytes was induced by PDGF-BB which also promoted αSMA (ACTA2) expression in the CNA.42^+^/NG2^+^ early differentiating FDCs. In contrast, TNF-α/LT-β substantially inhibited αSMA (ACTA2) and collagen type 1 (COL1A1) expression in CNA.42^+^/αSMA^+^ early FDCs compared to untreated cells. Furthermore, TNF-α/LT-β induced CD21/CR2 in CNA.42^+^/CR2^−^ immature, and FcγRIIB (CD32B) in mature CNA.42^+^/CR2^+^ FDCs.

Using immunocytochemistry, we further confirmed that treatment of the NG2^+^/αSMA^+^ type-1 pericytes with PDGF-BB induces CNA.42 expression. Cytospin preparations from the NG2^+^/αSMA^+^ population with and without PDGF-BB treatment were prepared and the mAb CNA.42 signal intensity was analyzed using Image J. As shown in Figure 4C, PDGF-BB significantly induced the FDC marker CNA.42 on the NG2^+^/αSMA^+^ cells compared to untreated and isotype-treated preparations.

Altogether, these results directly support the role of PDGF-BB in induction of the CNA.42^+^/αSMA^+^ early FDC phenotype from type-1 pericytes; and the capacity of TNF-α/LT-β to induce CR2 and FcγRIIB gene expression thus instating full maturation of FDCs.

### PDGF-BB induces pericyte CXCL13 expression and stromal cell migration

Mature FDCs are lacking in B cell-deficient microenvironments (31, 33) which emphasizes the need for B cell homing into lymphoid structures for effective FDC maturation. CXCL13, the B cell homing chemokine, is a typical product of mature FDCs, however, a different source of CXCL13 upstream of mature FDCs is required for B cell homing to proceed and induce FDC maturation. We have previously reported that CXCL13 is inducible upstream of FDC networks in the RA synovium (34) and, here, we sought to investigate the source of this CXCL13 and the effect of PDGF-BB on its expression.

**Figure 5:**
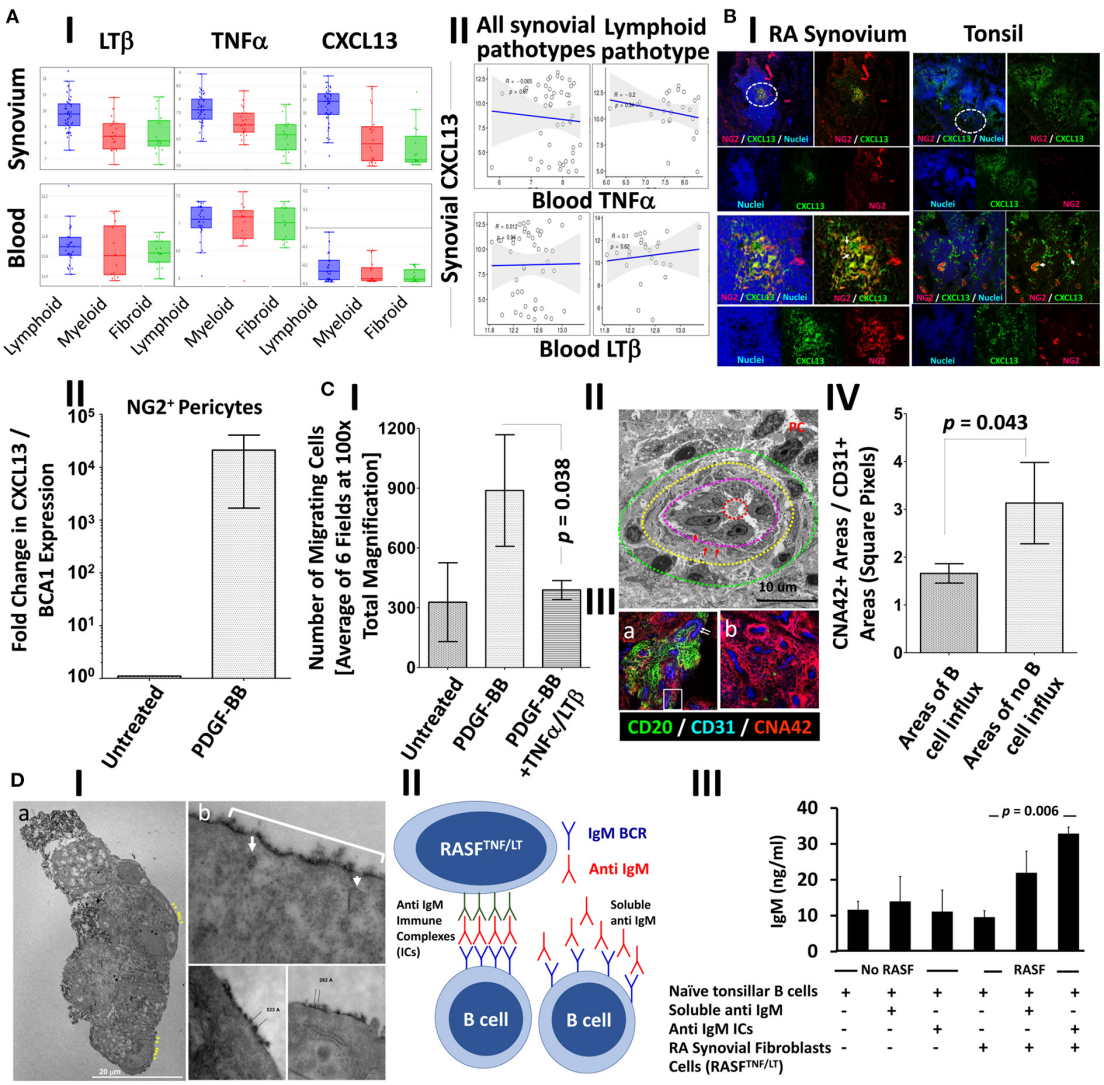
PDGF-BB-induces pericyte-CXCL13 secretion and RASF migration; and TNF-α/LT-β-activated RASF trap ICs and induce B cell activation. **(A)** RNA-Seq analysis of the PEAC cohort demonstrating the correlation of synovial and blood TNF-α, LT-β, and CXCL13 in the different RA synovial pathotypes **(A-I)**, and the correlation of synovial CXCL13 with serum TNF-α and LT-β in the synovial pathotypes in general and the lymphoid pathotype in specific **(A-II)**. **(B-I)** Immunohistochemical colocalization of CXCL13 (green) with NG2^+^ pericytes (red) [nuclei are stained blue] in the RA synovium and tonsils. Single, dual, and triple overlays are presented, and the areas surrounded by white circles are shown at higher magnification in the lower panel. **(B-II)** NG2^+^ pericytes were treated with 300 ng/ml PDGF-BB and the fold change in CXCL13 gene expression compared to untreated cells was calculated by Livak's DD equation and shown in bar graph. **(C)** Stromal/endothelial dissociation at the sites of B cell influx into the rheumatoid synovium. **(C-I)** The effect of PDGF-BB and TNF-α/LT-β on the trans well migration of RASF. Image J was used to process, segment, and automatically count the number of migrating RASF in 100× magnified fields after treatment with PDGF-BB with and without TNF-α/LT-β. The averages were calculated and displayed ± the SEM. **(C-II)** Transmission electron micrograph of the RA synovial HEVs showing concentric zones of the vascular lumen (dashed red circle), high cuboidal vascular lining (zone between the red and magenta dashed circles), multi-layered basement membrane (zone between the magenta and yellow dashed circles, red arrows), and zone showing mural cell displacement (between the yellow and green dashed circles). Plasma cell (PC) with cartwheel nucleus is seen adjacent to the HEV. **(C-III)** Immunohistochemical analysis showing the proportion of perivascular CNA.42 immature FDCs (red) to vascular CD31 (blue) at sites associated with (a) or devoid from (b) CD20^+^ B cells (green). The CD31^+^ endothelium in (a) is relatively of high phenotype [white arrows], and the white rectangle shows B cells insinuated between the endothelium and the CNA.42^+^ perivascular early FDCs. **(C-IV)** Image J quantification of the CNA.42 to CD31 ratio at the sites of B cell influx and no influx. **(D)** Induction of Ig secretion in *in vitro* GCRs supported by IC-loaded TNF-α/LT-β-stimulated RASF. **(D-I)** Transmission electron micrograph of HRP-loaded TNF-α/LT-β-stimulated RASF. The ultra-thin sections were left unstained to maximize visibility of the HRP-DAB deposition on the membranes. (a) A cluster of RA synovial fibroblasts showing surface deposition of the HRB substrate DAB (black, yellow arrows). (b) The periodic deposition of DAB (white bracket) is interrupted at certain sites (white arrows) due to internalization of the HRP antigen, and the distance between the antigen clusters range between 200 and 600Å. **(D-II)** Setup of BCR-mediated polyclonal B cell activation using anti-IgM BCR cross-linker. The anti IgM ICs formed of rabbit anti IgM Abs (green) + anti human IgM Abs (red) are loaded on the activated RA synovial fibroblasts to cross-link the IgM BCR (blue). Relatively, soluble anti human IgM Abs (red) do not effectively cross link the BCRs as they lack periodicity. **(D-III)** ELISA measurement of secreted human IgM in the culture supernatants at day 6. Data expressed as the mean ± SEM. Significance was calculated using 2-tailed unpaired student *T*-test and the *p*-value between soluble IgM and IgM ICs is shown.

Our RNA-Seq analysis indicated that synovial/local, but not blood/systemic, CXCL13, TNF-α, and LT-β expression strongly correlate with a lymphoid RA synovial pathotype (Figure 5A-I, Table 3). Moreover, blood TNF-α and LT-β did not significantly correlate with synovial CXCL13 expression whether analyzed in all synovial pathotypes or specifically in the lymphoid group (Figure 5A-II, Table 4). Consequently, we speculated that local sources of CXCL13, TNF-α and LT-β synergistically facilitate FDC-supported ectopic lymphoid neogenesis in the RA synovium. Our immunohistochemical studies indicated that CXCL13 colocalizes with NG2^+^ pericytes in the RA synovia, and this colocalization can also be seen in the tonsils (Figure 5B-I). Furthermore, stimulation of flow sorted NG2^+^ pericytes from tonsillar single cell suspensions with PDGF-BB induced upregulation of CXCL13 gene expression (Figure 5B-II).

**Table 3:** Correlation of synovial tissue and blood CXCL13, TNF-α, LT-β with RA synovial pathotypes.

		**Corrected** ***p*****-values**		
		**LTβ**	**TNFα**	**CXCL13**
Synovial	Lymphoid v myeloid	1.3e-05	6.1e-03	4.9e-03
	Myeloid v fibroid	7.5e-01	5.1e-02	2.8e-01
	Lymphoid v fibroid	1.9e-06	8.6e-08	2.2e-05
Blood	Lymphoid v myeloid	0.97	1.00	NA
	Myeloid v fibroid	0.78	0.95	NA
	Lymphoid v fibroid	0.32	NA	NA

**Table 4:** Correlation of synovial tissue CXCL13 with blood TNF-α and LT-β in RA synovial pathotypes.

	**All pathotypes**	**Lymphoid pathotype only**
Blood TNFα vs. synovial CXCL13	R = −0.065 *p* = 0.67	R = −0.2 *p* = 0.34
Blood LTβ vs. synovial CXCL13	R = 0.012 *p* = 0.94	R = 0.1 *p* = 0.62

Chemokine-guided leukocyte influx, including CXCL13-mediated B cell homing, in inflamed tissues is promoted by stromal cell migration clear of the vascular endothelium, thus, generating permissive sites devoid of mural coverage (35), and we argued that PDGF-BB may also contribute to this process in rheumatoid synovitis. In fact, PDGF-BB significantly stimulated RA synovial stromal cell migration above baseline, whereas co-treatment with PDGF-BB and TNF-α/LT-β inhibited the PDGF-BB-induced migration (Figure 5C-I). Distancing between the stromal support and the lining of the HEVs was evident by TEM, and this stromal/endothelial separation was also associated with extensive multilayering of the vascular basement membrane suggestive of substantial remodeling by transmigrating cells as previously reported (36) (Figure 5C-II). Moreover, the density of the CNA.42^+^ perivascular FDCs [relative to the CD31^+^ vascular endothelium] was significantly less at the sites of CD20^+^ B cell influx indicative of stromal/endothelial dissociation (Figures 5C-III,IV).

In summary, by facilitating B cell influx through induction of CXCL13 and stromal cell dissociation, PDGF-BB contributes to FDC maturation in the RA synovium.

### TNF-α/LT-β-activated RA synovial fibroblasts (RASF) trap ICs and induce T cell-independent B cell activation in *in vitro* germinal center reactions

B cell activation by FDC-retained ICs is a cardinal feature of mature FDCs. We have demonstrated that TNF-α/LT-β upregulated the expression of the IC-retaining receptors CR1, CR2, and FcγRIIB (Figures 3F–H), and here we sought to test whether TNF-α/LT-β-activated RASF could functionally trap ICs and activate B cells.

To investigate this, we first loaded TNF-α/LT-β-stimulated RASF with HRP ICs and assessed the retention pattern of the HRP substrate diaminobenzidine (DAB) using TEM as previously detailed (37). Our results demonstrated that DAB was seen on the cell membrane and dendrites emanating from them (Figure 5D-Ia), and the ICs were periodically arranged with 200–600 Å spacing between epitopes that fits our reported range of periodicity optimum for BCR crosslinking (5, 7). It was also noticeable that the periodicity on the plasma membrane is interrupted in certain areas due to internalization of the ICs (Figure 5D-Ib) which may be related to receptor recycling as previously described (38).

We then assessed the ability of activated RASF to deliver strong BCR-mediated signal by cross linking the BCRs and induce antibody secretion independent of T cell help. To do this, we set up *in vitro* GCRs where untouched tonsillar B cells were stimulated with anti IgM in the form of ICs as we previously described for the mouse B cells using anti IgD ICs (5), [diagrammatically represented in Figure 5D-II]. This system uniquely allows polyclonal B cell stimulation in a BCR-dependent manner and produces ELISA-measurable Ig level after subtraction of background levels that may register due to cross reactivity with the used ICs. As shown in Figure 5D-III, 6 days after culture, the stimulated RASF promoted production of higher levels of IgM and the IC-loaded cells were more efficient in this induction than soluble IgM.

Together, our data imply that TNF-α/LT-β-activated RASF periodically retain ICs and induce T cell independent B cell activation and IgM production in *in vitro* GCRs.

### Peptidyl citrulline retention on RA synovial FDCs and correlation of the FDC genes module with the serological, cellular, and clinical parameters of RA

**Figure 6:**
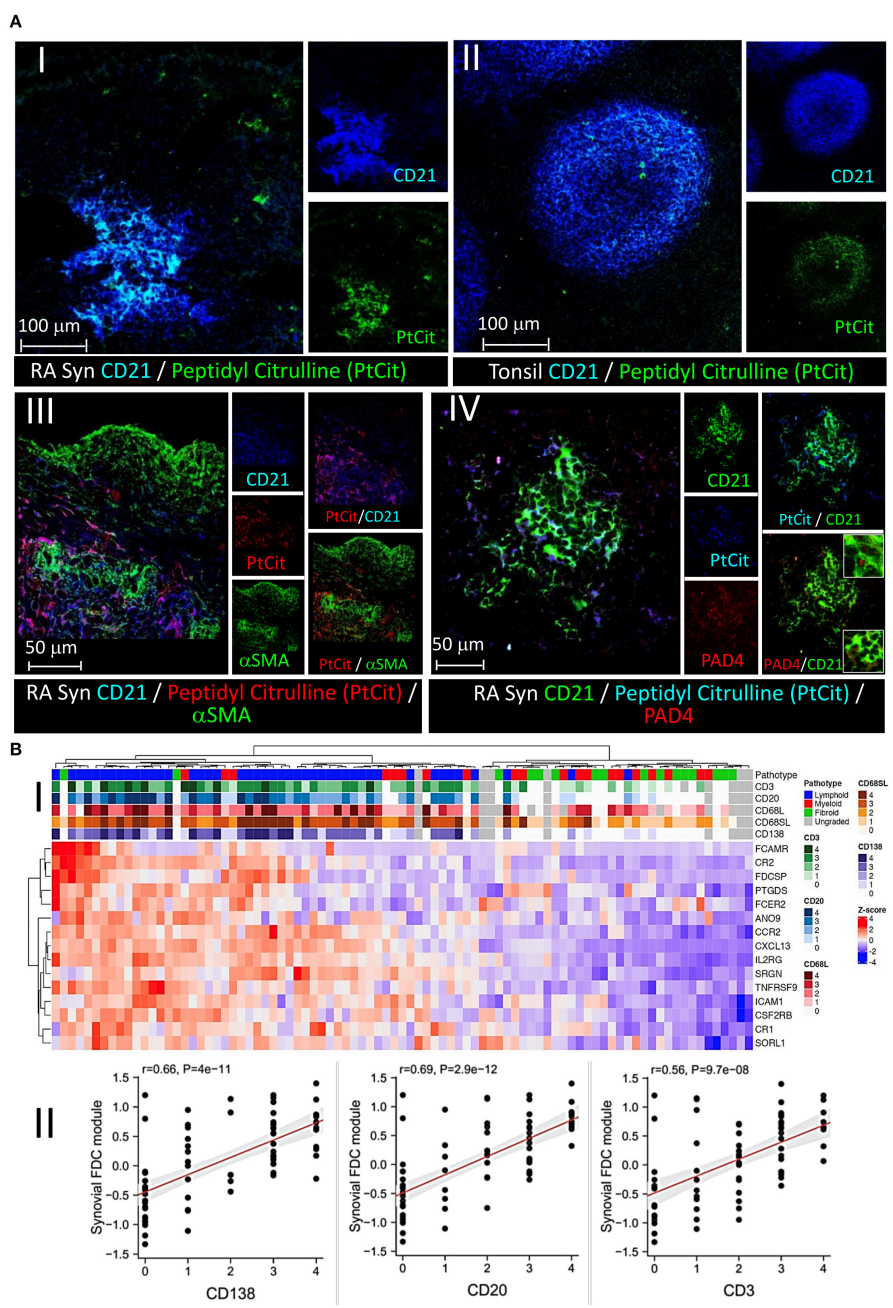
Retention of peptidyl citrulline on synovial FDCs and correlation of the FDC gene module with synovial pathotypes and cellular infiltrates**. (A)** Colocalization of peptidyl citrulline with FDCs in the synovial tissues **(A-I)**, and tonsillar control **(A-II)**. **(A-III,IV)** peptidyl citrulline trapping on CD21^+^ synovial FDCs but not CD21^−^/αSMA^+^ myofibroblasts **(A-III)**, and colocalization of PAD4 with CD21^+^ synovial FDCs and peptidyl citrulline [**(A-IV)**, insets: Upper intracellular PAD4, Lower, extracellular PAD4]. Scale bars, overlays and single channels are shown with the relevant panels. **(B)** Correlation of the FDC module genes with synovial pathotypes and cellular infiltrates. **(B-I)** Heatmap showing hierarchical clustering of the synovial FDC module gene expression (variance stabilizing transformed counts) against different synovial pathotypes. Upper tracks show histological scores for CD3, CD20, CD68L (lining), CD68SL (sublining), and CD138 and overall pathotype. Tracks on the left-hand side indicate the relationships between the FDC genes expression across all the pathotypes. **(B-II)** Correlation of the synovial FDC gene module with synovial plasma cells (CD138), B cells (CD20), and T cells (CD3). r = Spearman rho correlation coefficient, *p* = significance value.

Anticyclic citrullinated peptide (CCPs) antibodies targeting citrullinated proteins are the hallmark of RA. We have previously shown that FDC^+^ synovial ELS harbor CD138^+^ plasma cells immunoreactive with citrullinated antigens (1). Here, we sought to directly confirm retention of citrullinated proteins on synovial FDCs and colocalize them with FDC markers. As shown in Figure 6A, CD21^+^ synovial (i) and tonsillar FDC (ii) reticula colocalize with mAb (clone F95) that specifically recognizes peptidyl- but not free citrulline. Importantly, this retention was associated with emergence of CR2 (CD21) on early FDCs but not on their αSMA^+^/CR2^−^ precursors (iii). Interestingly, synovial FDCs express intracellular and secreted PAD4 in association with the trapped citrullinated proteins (iv).

To further explore the association of FDCs with the different types of rheumatoid synovitis and cellular composition, first, we constructed a 15-gene FDC module comprising genes that are highly expressed in FDCs and differentiate them from other stromal cells, as recently reported by single cell RNA Seq and microarray studies (39). The module included FDC-related antigen retaining receptors [CR1, CR2, FcαμR, FcεR2], secreted factors and adhesion molecules [CXCL13, FDC secreted protein (FDCSP), Serglycin (SRGN) and ICAM1], as well as cytokine, chemokine, and growth factor receptors [TNFRSF9, CCR2, CSF2RB and IL2-R]. Next, we correlated the FDC genes module with the synovial pathotypes and specific cell lineages infiltration. As shown in Figure 6B-I, high expression scores of the individual FDC genes clustered with the lymphoid pathotype and high histological scores of CD138 plasma cell, CD20 B cell, and CD3 T cell infiltration. This association was further supported by the strong correlation of the overall synovial FDC module expression with the histology scores of the infiltrating cells (Figure 6B-II). The high histology scores of CD68 macrophages in the lymphoid pathotype agrees with the lymphoid/myeloid overlap defined in the lympho-myeloid pathotype.

**Figure 7:**
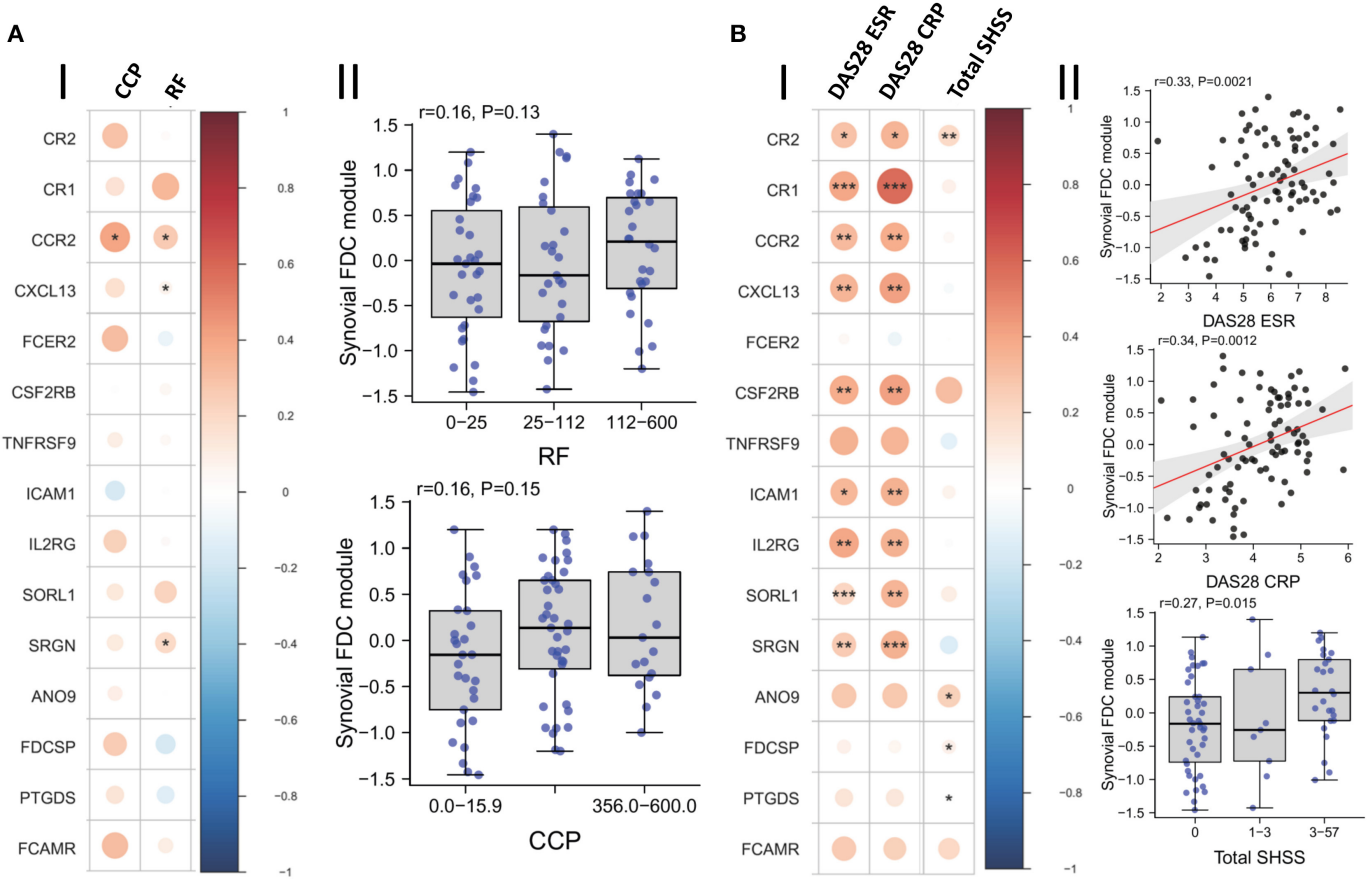
Correlation of synovial FDCs with the RA clinical parameters: **(A-I)** Correlation plot of the individual FDC genes against baseline CCP (anti-cyclic citrullinated peptide antibody titer) and RF (rheumatoid factor titer). **(A-II)** Boxplots demonstrating the correlation of RF and CCP titers with the overall synovial FDC genes module. **(B-I)** Correlation plot of the individual FDC genes against DAS28-ESR (28-joint Disease Activity Score for rheumatoid arthritis with Erythrocyte Sedimentation Rate); DAS28-CRP (C Reactive Protein), and Sharp van der Heijde score (SHSS). **(B-II)** Correlation of DAS28-ESR; DAS28-CRP, and SHSS with the overall synovial FDC genes module. *p*-values were calculated using Spearman rank test, *P < 0.05, **P < 0.01, ***P < 0.001. r = Spearman rho correlation coefficient, *p* = significance value.

Moreover, we investigated the relationship between FDC positivity and seropositivity; and our results (Figure 7A) demonstrate that while the FDC module positively correlates with the anti CCP and RF titres, and some genes like CXCL13, CCR2, and SRGN (Serglycin) are significantly associated with seropositivity, the overall correlation did not reach significance.

Nevertheless, local production of high affinity class switched autoantibodies is FDC-dependent and contributes to increased disease severity and tissue damage (3). Indeed, our results in Figure 7B show strong correlation between the FDC module, disease activity scores, and radiographic features of tissue damage as assessed by the Sharp/van der Heijde scores.

### Correlation of IL-6 with synovial FDCs and RA pathotypes

**Figure 8:**
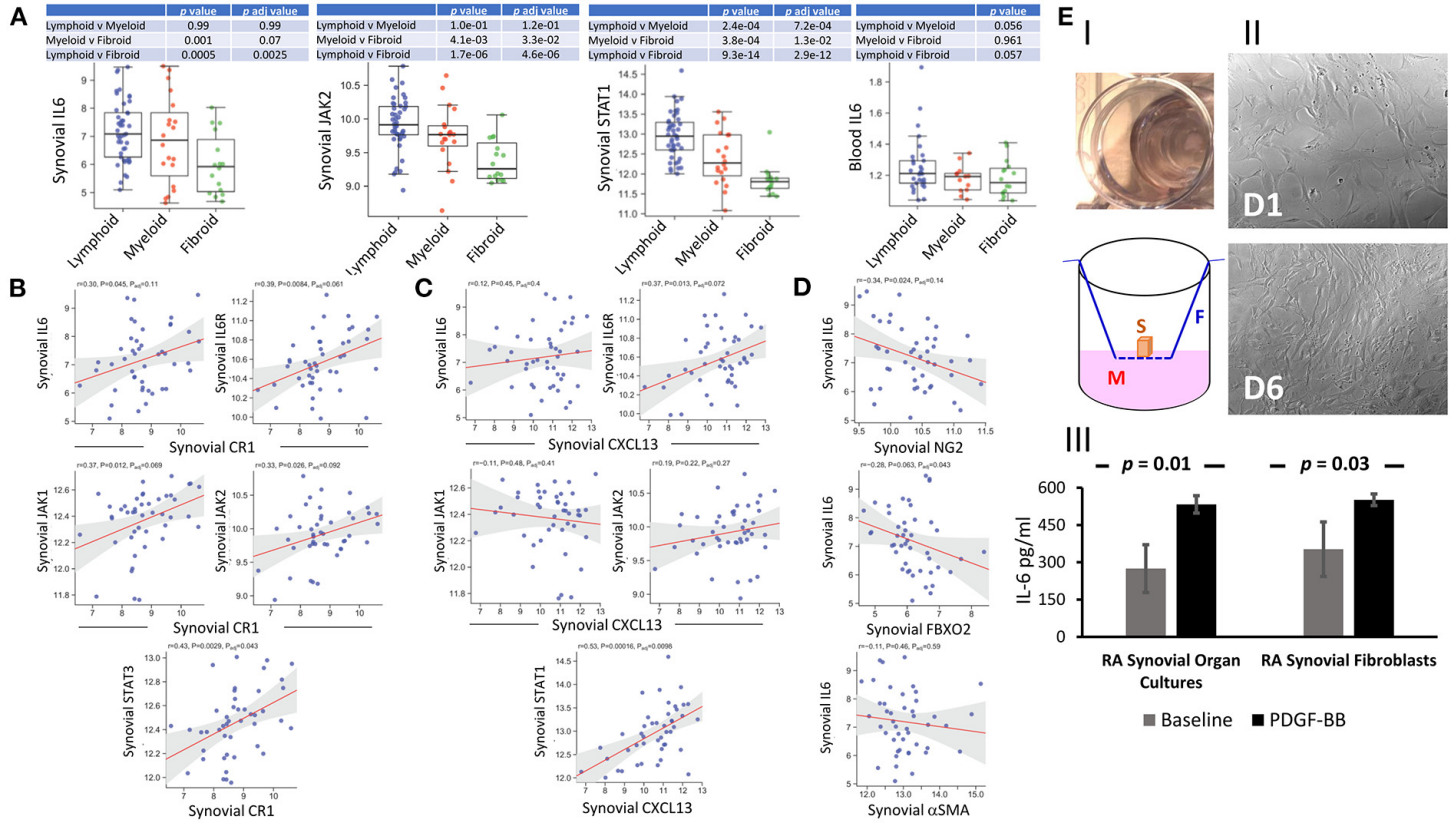
Correlation of IL-6 expression with synovial pathotypes and FDC markers. **(A)** Boxplots displaying the correlation of synovial IL-6, JAK2, STAT1, and blood IL-6 expression with synovial pathotypes. **(B,C)** Correlation blots of synovial IL-6, IL-6R, JAK1, and JAK2 expression with the FDC markers complement receptor 1 (CR1/CD35) and CXCL13, respectively. Correlation of STAT3 and STAT1 with CR1 and CXCL13 respectively are also shown. **(D)** Correlation of synovial IL-6 expression with the markers associated with early FDC differentiation including NG2 (pericytes), αSMA (myofibroblasts), and FBXO2 (CNA.42). **(E)** IL-6 release from synovial organ and fibroblast cultures stimulated with 300 ng/ml PDGF-BB for 24 hrs and 6 days respectively. **(E-I)** Synovial organ culture showing a piece of synovial tissue placed in cell culture inserts mounted in 24-well plates (Upper). Diagrammatic representation of the synovial organ culture setup (S = Synovial Tissue, M = Culture Medium, F = Filter Device). **(E-II)** Rheumatoid arthritis synovial fibroblasts (RASFs) at base line (Day 1 = D1) and after 6-day (D6) stimulation with PDGF-BB. IL-6 levels at baseline and after stimulation are shown in **(E-III)**. Cultures were run in triplicates and data is expressed as the mean ± SEM. Significance was calculated using 2-tailed unpaired student T test and the *p*-value between baseline and PDGF-BB stimulation is shown.

FDC-IL-6 is critically involved in productive GC reactions; and the absence of FDC-IL-6 correlates with a reduction in GCs, SHM, CSR, and antigen-specific antibodies (40). Here we sought to investigate the correlation of synovial tissue and blood expression of IL-6 with synovial pathotypes and FDC markers. IL-6/IL-6R/gp130 signal vial Jak1, Jak2 and Tyk2/STAT1, STAT3 and to a lesser extent STAT5 (41). Our results (Figure 8A) show significant correlation between synovial IL-6, JAK2, STAT1; blood IL-6 and lympho-myeloid synovitis. Furthermore, IL-6, IL-6R, JAK1, and JAK2 significantly correlated with the mature FDC markers CD35/CR1 (Figure 8B) and CXCL13 (Figure 8C). Moreover, STAT3 and STAT1 strongly correlated with CD35/CR1 and CXCL13, respectively. On the other hand, synovial IL-6 inversely correlated with genes associated with immature FDCs including NG2 (pericytes), αSMA (myofibroblasts), and FBXO2 (CNA.42) (Figure 8D). These correlations suggest that IL-6 is associated with mature synovial FDC phenotype, and consequently functions, involved in effective GC reactions. Additionally, we sought to see whether PDGF-BB, which is associated with early FDC differentiation, could induce IL-6 in synovial organ and fibroblast cultures. As indicated in Figure 8E; synovial organ (i) and fibroblast (ii) cultures stimulated with 300 ng/ml PDGF-BB for 24 h and 6 days, respectively, showed significantly higher levels of IL-6 that increased from a mean of 275 to 533 pg/ml [RA synovial fluid IL-6 = 1,365 pg/ml + 1,850 (42)].

### The mAb CNA.42 recognizes F-box only protein 2 (FBXO2)

The unique ability of the CNA.42 mAb to identify immature and mature forms of FDCs with comparable efficiency in both SLTs and RA ELSs prompted our search for the antigen recognized by this mAb using tonsils as an ample source of the antigen.

**Figure 9:**
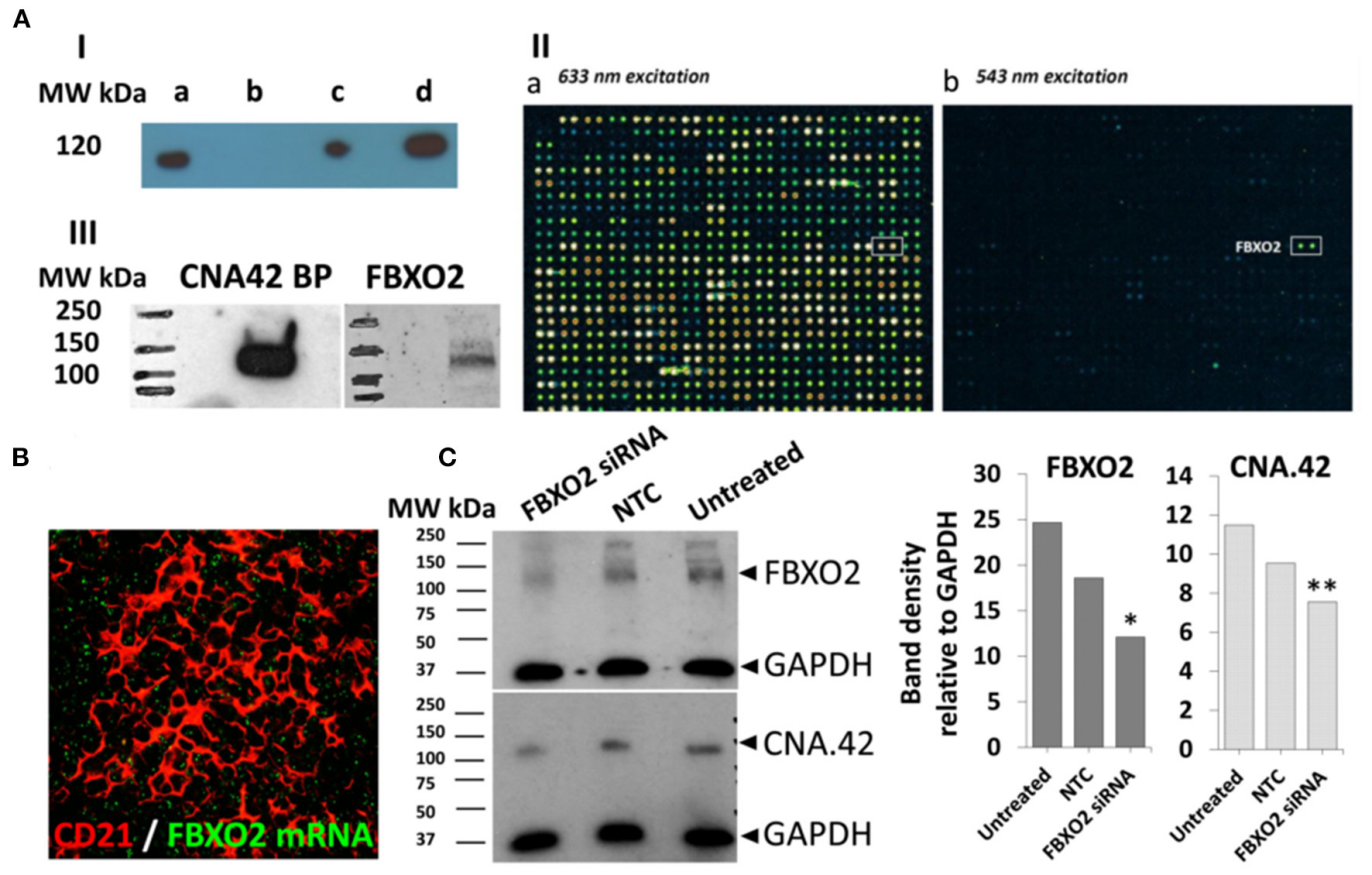
The FDC mAb CNA.42 recognizes FBXO2. **(A)** Immunoprecipitation (IP) and characterization of the CNA-42 binding protein. **(A-I)** Western blotting of total cell lysate (a), negative control (agarose beads only) and the CNA.42-immunoprecipitated proteins (c and d, 1.5 and 6 uls/lane respectively) from tonsillar single cell suspension probed with CNA-42. A single band is detectable at 120 Kd. **(A-II)** the reactivity of the CNA.42 mAb on the HuProt™ human proteome microarray showing subarray 9-1 of array 1300017931 (used for the CNA.42) with fluorescence detection at 633 nm excitation (a) and 543 nm excitation (b). (a) Staining with biotinylated anti-GST and Streptavidin-647. Rows 1-28 show generic staining of the GST-tagged immobilized human proteins, among them FBXO2 in row 11. (b) Probing with CNA.42 and Cy3 labeled anti-mouse IgM shows one hit, the human protein FBXO2 in the subarray. **(A-III)** Western blotting of tonsillar lysates with FBXO2 and CNA-42-specific antibodies recognize 120 Kd bands in the lysates [CNA.42 BP = CNA.42 binding protein]. **(B)** *In situ* hybridization of FBXO2 mRNA (green) showing intracellular signal in tonsillar CD21^+^ FDC reticula (red). **(C)** Western blotting of lysates from the CAN.42 expressing CEM cell line using mAb CAN.42 and anti FBXO2. CEM were untreated or treated either with Accell human FBXO2 siRNA (1 uM), or non-targeting control (NTC). GAPDH is used as a loading control. Compared to untreated cells, densitometric analysis with Image J indicates that FBXO2 siRNA-treated cells expressed 50% (*) and 35% (**) less FBXO2 and CNA.42, respectively.

First, we immunoprecipitated the target protein (Figure 9A-I) from the cell membrane fractions of tonsillar single cell suspensions and subjected it to analysis by mass spectrometry, however, the results from different facilities were inconsistent.

We then assessed the reactivity of the mAb on human proteome profiler (Figure 9A-II) hosting ~20,000 proteins, and a single positive signal [with a hit score of 15.2 and interaction score of 3.1] recognizing F-box Only Protein 2 (FBXO2), a ubiquitin ligase, was identified. Using FBXO2 specific Ab, we identified a 120 Kd band (Figure 9A-III) in tonsillar lysate at the same MW identified by the mAb CNA.42.

While the reactivity of the commercially available mAbs against FBXO2 was sufficient to detect the target band in WBs, they failed to provide consistent data in the tissues which may be related to protein expression below the sensitivity of immunohistochemistry. So, we assessed FBXO2 mRNA expression by *in situ* hybridization, and the FBXO2 mRNA was seen in the CD21^+^ FDC reticula in tonsillar sections (Figure 9B). Furthermore, using siRNA, we knocked down FBXO2 in the FBXO2-expressing CEM/C2 cell line and, compared FBXO2 and CNA.42 expression in treated and untreated cells. As illustrated in Figure 9C, siRNA-treated cells expressed 50 and 35% less FBXO2 and CNA.42, respectively.

Overall, these studies suggest that the CNA.42 mAb binds FBXO2 and its expression can be interfered with using FBXO2-specific siRNA.

## Discussion

FDCs substantially contribute to ELS neogenesis in RA which correlates with progressive bone erosion, joint destruction, and poor prognosis. Compared to the predetermined nature of cell development in SLT, the evolving microenvirnment in RA ELS allows multi- and cross-destination fates of cellular ontogeny. Herein, we systemically investigated the role of PDGF-BB and TNF-α/LT-β in FDC development and pathotype differentiation of RA synovitis; and we report that: (1) Early FDCs in the RA synovial perivascular space display the pericyte and fibroblast markers PDGFR-β, NG2, and Thy-1 which are lost in mature FDCs supporting well-developed ELSs. (2) Bulk RNA-Seq analysis of RA synovial biopsies shows positive correlation of PDGF-BB/PDGFR-β and TNF-α/LT-β expression with genes related to early FDCs/fibroid and mature FDCs/lymphoid RA synovial pathotypes, respectively. (3) *In vitro* stimulation of synovial stromal cells with PDGF-BB and TNF-α/LT-β induces early and late FDC-related genes, respectively. (4) *In vitro* activation of sorted stromal cell subsets with PDGF-BB induces the CNA.42^+^/CR2^−^ early FDC phenotype from NG2^+^/αSMA^+^ type-1 pericytes; while TNF-α/LT-β induces the maturation markers CR2/CD21 and FcγRIIB in CNA.42^+^ FDCs (5) PDGF-BB promotes migration and CXCL13 expression in RASF and pericytes, respectively. (6) TNF-α/LT-β-activated RASF periodically display antigens as ICs and stimulate T cell independent B cell activation in *in vitro* GCRs. (7) The FDC mAb CNA.42 recognizes FBXO2, a subunit of an E3 ubiquitin ligase complex.

**Figure 10:**
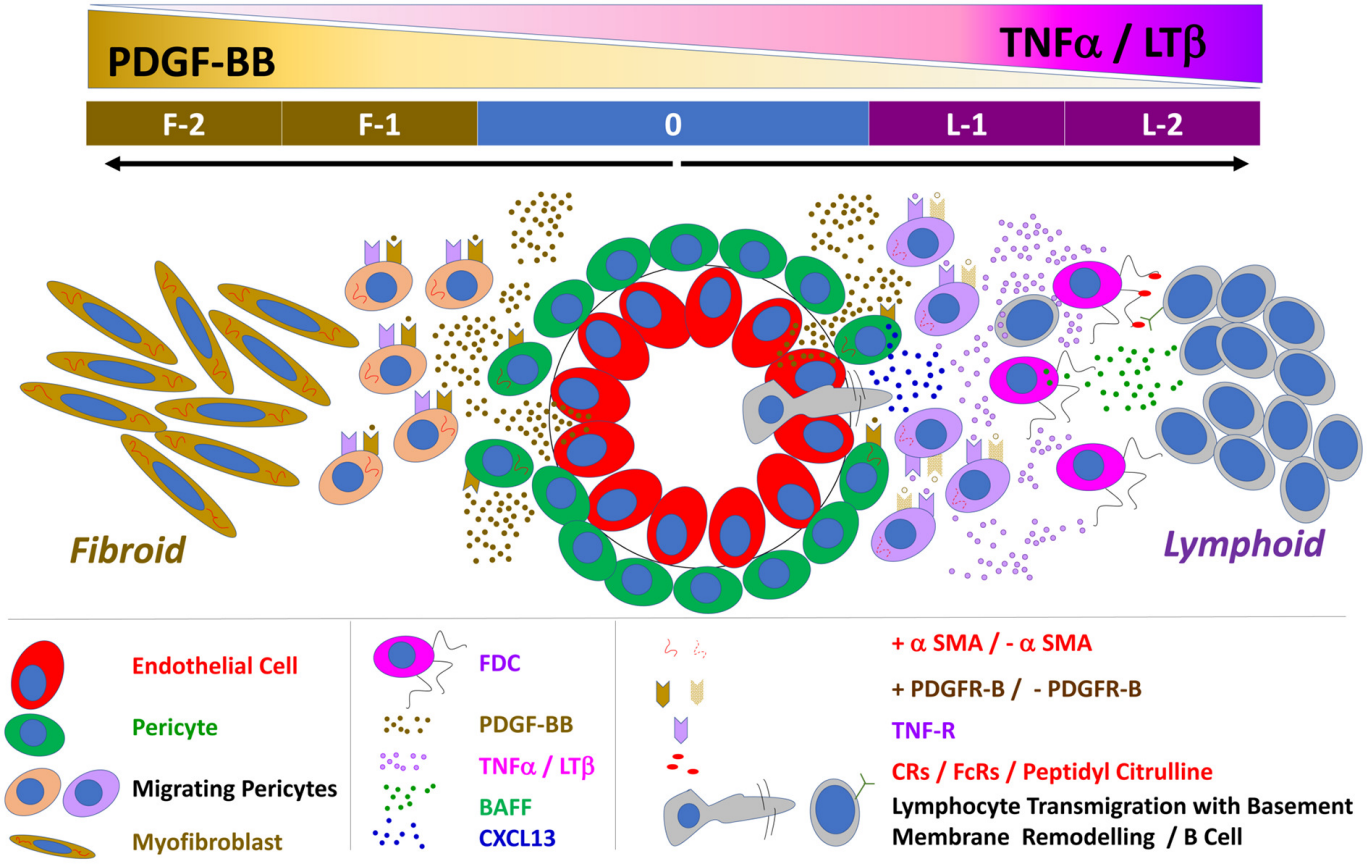
Schematic representation of the proposed interaction between PDGF-BB and TNF-α/LT-β in FDC development and induction of lymphoid vs. fibroid RA pathotypes: 0 = Neo-angiogenesis in the inflamed synovium is regulated by the reciprocal activation of the endothelial cells and their covering pericytes. PDGF-BB is mainly produced by endothelial cells and activates the PDGFR-β ^+^ pericytes whereas pericytes are the main source of VEGF that stimulates endothelial growth and sprouting. PDGF-BB induces αSMA expression, pericyte migration, TNF-R expression, and the FDC marker CNA.42 on the ubiquitously available perivascular FDC precursors. F-1 = In the absence of TNF-α/LT-β, the TNF-R is not engaged, and the effect of PDGF-BB is maintained leading to extensive generation of αSMA^+^ myofibroblasts. F-2 = Myofibroblasts further divide, migrate and invade bones and cartilage, the vasculature degenerates due to loss of the stimulatory effect of the pericyte-derived VEGF and a fibroid pathotype of RA synovitis is instituted. L-1 = Pericyte activation by PDGF-BB induces CXCL13 expression and dissociation from the vessel wall thus facilitating B cell influx which extensively remodel of the vascular basement membrane. In the presence of B cell derived TNF-α/LT-β, the TNF-R is engaged leading to (1) downregulation of PDGFR-β and αSMA expression (2) upregulation of the FDC maturation markers like the antigen retaining complement and Fc receptors and BAFF. L-2 = Synovial retention of the circulating auto ICs on the mature FDC reticula induces B cell activation, proliferation, and local autoantibody production, thus establishing a fully functional ectopic lymphoid-like structure.

Our proposed model of FDC maturation and pathotype differentiation in the RA synovium (Figure 10) begins with mutual activation of endothelial cells and pericytes *via* VEGF and PDGF-BB, respectively. PDGF-BB-activated pericytes (1) differentiate into CNA.42^+^ early FDCs, (2) secrete CXCL13, and (3) express TNF-αR. In the presence of high synovial levels of TNF-α/LT-β (1) PDGFR-β is downregulated on the early differentiating FDCs, (2) endothelial cells acquire a HEV phenotype, (3) lymphocytes are recruited to the synovium guided by the pericyte-derived CXCL13 gradient, and (4) lymphocyte derived TNF-α/LT-β induce full maturation of FDCs *via* the TNF-α/LT-β receptors. Mature FDCs retaining auto-antigens and secreting CXCL13 further recruit and activate B cells, thus, instituting immunologically active ELSs. Conversely, in the absence of high synovial levels of TNF-α/LT-β (1) PDGFR-β expression remains high on the CNA.42^+^ early FDCs (2) PDGF-BB induces FDC to myofibroblast transition, and (3) activated myofibroblasts extensively divide, migrate, and invade bone and cartilage, thus, establishing fibroid synovitis. The high local levels of TNF-α/LT-β in the rheumatoid synovium before leukocyte influx could be attributed to resident synoviocytes which respond to various stimuli by secreting proinflammatory cytokines (43, 44).

The role of PDGF-BB/TNF-α/LT-β interaction in driving synovial fibrosis and lymphoid neogenesis, as proposed in our model, is consistent with clinical studies reporting the effect of anti-TNF-α on the rheumatoid synovium. TNF-α blockade in RA patients results in drastic attenuation of FDC reticula and ELSs together with extensive sublining fibrosis, synoviocyte detachment and loss of the vasculature characteristic of fibroid synovitis (45, 46).

Mature FDC reticula are stationary niches for antigen display, affinity maturation, and memory B cell generation. These features agree with our results on the critical role of TNF-α/LT-β in late FDC differentiation where they upregulate the antigen retaining receptors and downregulate αSMA expression and stromal cell migration. In fact, consistent with our data; the ability of TNF-α to inhibit αSMA expression and stromal cell migration has been reported in several studies investigating human synovial fibroblasts (47), joint capsule myofibroblasts (48) dermal fibroblasts (49, 50), and human gingival fibroblasts (51). Interestingly, this inhibitory effect continues after removal of TNF-α indicating a persistent functional change, most probably loss of the αSMA protein (52).

The restricted emergence of mature FDC reticula in synovial ELSs despite the unrestricted availability of perivascular FDC progenitors argues against systemic control of FDC differentiation and implies local regulation. In fact, our RNA-Seq data indicated that synovial tissue, but not blood, CXCL13, TNF-α, and LT-β significantly correlated with RA lymphoid synovitis. While our correlations were derived at an RNA level, proteins may follow the same pattern, and actually, it has been reported that the protein levels of TNF-α and IL-6 in the synovial fluid were not significantly different in patients with ELS^+^ and ELS^−^ RA synovitis (53).

The shared phenotype of pericytes, fibroblasts and early FDCs in the synovial perivascular spaces reflects the exceptional overlap of stromal cell subsets. Indeed, pericytes, mesenchymal stromal/stem cells, smooth muscle cells, fibroblasts and myofibroblasts are phenotypically overlapping and their trans-differentiation has been extensively studied (54–60). Indeed, it has been recently shown that positional identity of mesenchymal cells follows specific transcriptional gradients in the RA synovium (61), and this may partly explain acquisition of different functions at different locations by cells that share common ancestry.

Our data agree with a mesenchymal origin of RA synovial FDCs, nevertheless, mature FDCs in established ELSs (as well as SLTs) lack expression of specific mesenchymal markers including Thy-1 and extensively express markers like CD21 which are deficient in mesenchymal cells (62). Consequently, earlier studies suggesting that FDCs are a specialized mesenchymal cell population within germinal centers (63) should be interpreted in the context of the developmental phases of FDC differentiation where mesenchymal signatures correlate mainly with the early perivascular but not the mature FDC phenotype. It is also worth mentioning that, fully mature FDCs lack surface adherence and forms floating clusters in cultures (64–67) whereas their stromal precursors are typically adherent. We have used adherent synovial and tonsillar stromal cells in our *in vitro* assays to exclude mature FDCs from the cultures, and thus, delineating the early mechanisms of FDC differentiation from their precursors.

Remarkably, CD21 was expressed on early differentiating FDCs transitioning from NG2^+^/CNA.42^+^ to CNA.42^+^/CD21^+^ phenotype. Early acquisition of CD21/CR2 by the developing FDCs agrees with previous studies reporting that CRs are the earliest antigen retaining receptors to be expressed by FDCs (68) followed by the FcγRs that are induced upon IC binding to the CRs (69). Further maturation of FDCs and expression of FDC-FcγRIIB, ICAM-1 and VCAM-1 is induced by IC binding to the FcRs in an autocrine fashion (70).

The ability of PDGF-BB to stimulate stromal cell migration dissociation from the vessel, thus granting access to B cell entry, seems to be crucial to institute a lymphoid pathotype supported by mature FDC reticula. In fact, FDC maturation requires only a small number of mature B cells (68) and our results indicate that the areas of B cell influx have less CNA-42^+^ early FDCs than areas of no influx when compared to the vascular areas suggestive of early FDC displacement.

The role of synovial FDCs in promoting local auto-antibody production and class switching has been substantiated in several studies reported by our group and others. We have shown that AID expression is limited to FDC^+^ ELS where RA synovial plasma cells display immunoreactivity against citrullinated antigens. Moreover, engraftment of ELS^+^ (but not ELS^−^) RA synovial tissue into SCID mice resulted in the release of human class-switched ACPAs into the mouse circulation (1). Importantly, the ACPA response was RA specific since engraftment of ELS^+^ salivary glands' tissues from patients with Sjögren syndrome leads to the release of anti-Ro/SSA and anti-La/SSB human IgG but not ACPAs (71). The lack of significant correlation between the FDC module and seropositivity in our results is not surprising as although we previously reported that seropositivity significantly correlated with synovial lymphoid and plasma cell-associated gene expression (3, 4, 72), synovial FDC^+^ lymphoid structures with GC function represent a subset (~25–29%) of lymphoid aggregates characterized by large, more organized lymphoid like structures (Grade 3) (1, 34, 73). Thus, not all synovial lymphoid aggregates are synovial FDC positive, likely to explain the statistical discrepancy with previous publications. It is worth noting that, synovial contribution to seropositivity does not rule out autoantibody production in SLT particularly in the pre-arthritic phase when the synovium is normal (74–76). In fact, IgM RF could be detected in 100% of lymph nodes taken from seropositive patients but not normal controls (77); and lymph node B cell numbers tend to increase in these patients (78).

The list of potential native and modified intracellular and extracellular antigens that could be trapped on synovial FDCs is unlimited, which restricts the utility of imaging in identifying the specific proteins retained on FDCs. Nevertheless, using single B cell cloning, we have reported that ~40% of the synovial B-cell response in TLS^+^ RA patients is directed toward citrullinated antigens including histones, fibrinogen, and vimentin (79) which provides an indirect indicator of the nature of few autoantigens on FDCs. However, antigen retention on FDCs is a dynamic process (80), and through processes like epitope spreading (81–84), we reason that synovial FDC-archived autoantigens could be periodically updated, thus, promoting newer autoantibody production and disease progression. Furthermore, PAD4 expression by FDCs may contribute to citrullination of FDC trapped proteins and antigens released from apoptotic cells during the GCR, thus, further diversifying the nature of FDC-retained autoantigens.

The significant correlation between synovial FDCs, lympho-myeloid synovitis, and the expression of IL-6, IL-6R and its signaling pathway, as illustrated in our results, agrees with the critical role of FDC-IL-6 in promoting GCRs and somatic hypermutation leading to affinity maturation as previously reported in SLT (40). In addition, the ability of PDGF-BB to induce IL-6 in synovial organ and fibroblast cultures suggests that PDGF-BB could also contribute to the maintenance of mature FDC structure and function over and above its role in early FDC differentiation.

The influence of synovial pathotypes on RA patients' response to biologics has been described in our recent publications. In two studies, we have reported the response of RA patients to TNF inhibitors and the correlation of this response to different synovial pathotypes. In one study (85), 67.6% of patients responded to 12 weeks anti-TNF treatment with DAS28 fall >1.2 (ΔDAS28 response). The response was significantly [*p* = 0.002] associated with a lympho-myeloid and diffuse-myeloid pathotype, suggesting the possibility of modulating lymphoid aggregates. This is supported by the change in DAS28 associated with differences in the CD20^+^ B-cell scores between baseline and 12-weeks post-anti-TNF treatment (r = 0.66, *p* < 0.05), suggesting that clinical improvement and responsiveness to anti-TNF is associated with a reduction in the size of synovial B cells critical for the formation of lymphoid aggregates. In another report (86), we have shown that RA patients with robust responses to anti-TNF therapies are characterized at baseline by immune pathway activation involving lymphocytes, memory B cells, and plasmablasts; and that these pathways were inhibited by anti-TNF treatment both at the synovial gene expression and peripheral proteins levels. While the direct effect of anti-TNF treatment on synovial FDCs was not the objective of these studies, FDC maturation and maintenance requires membrane-bound signals from lymphocytes, and it is plausible that the reported reduction in the size of synovial B cell aggregates in anti-TNF treated patients could impact the integrity and function of FDC reticula. Studies investigating the effect of anti-TNF on SLT (tonsils) showed that anti-TNF treatment inhibits GC reactions and FDC networks as labeled with the CNA.42 FDC-specific Ab (45). Whether anti-TNF therapy directly or indirectly affects synovial FDC structure and function; and whether the impact of anti-TNF is different in SLT and TLS remain to be elucidated.

FDC reticular disintegration/persistence in the rheumatoid synovia after different treatments could represent potential mechanisms of drug response/resistance and/or disease control/relapse. Given the small number of FDC^+^/GC^+^ biopsies (~5%), at the 16-week primary end point of 2nd biopsies, in our current clinical trials, it was difficult to demonstrate the impact of various therapeutics on synovial FDCs and draw direct conclusions. Nevertheless, our published studies from the R4RA clinical trial indicated that: (1) Molecular stratification of the RA synovial tissues according to the B cell signature demonstrated that rituximab and tocilizumab are comparably effective in anti-TNF-inadequate responders in the B-cell rich population (87); (2) In paired synovial samples taken at baseline and 16 weeks post-treatment, (a) histologically, B cells and plasma cells were significantly reduced in patients treated with rituximab but not tocilizumab, (b) Comparing changes in gene expression over time; (i) rituximab-responders showed significant decrease in Ig and CD20 genes expression together with significant reduction in the FDC-relevant CXCL13 expression (ii) tocilizumab-responders showed significant reduction in IL-6- and lymphoid follicle-related transcripts including the FDC-related CR2 (88). The effect of rituximab and tocilizumab on the lymphoid architecture of the rheumatoid synovium, could subsequently affect the structural and functional properties of FDCs. However, as said, the above reports are limited by the small number of FDC^+^/GC^+^ biopsies at the 16-week time point, which may not be long enough to reveal the effect of biologics on stromal cells including FDCs and/or may not have sufficient power. A well-powered FDC-focused study would be required to investigate the effect of RA therapeutics on FDCs. Histological assessment of the FDC reticular size rather than lymphoid aggregates in paired synovial samples is needed to directly assess the effect of treatments on the FDC structure. Molecular tracing of the synovial FDC module, at the gene and protein levels, pre and post-treatment will also be required and the effect of therapies on early and late FDC differentiation will help identifying novel targets for drug resistant RA patients. We are planning to investigate these dimensions using synovial biopsies from RA patients recruited to the current and future clinical trials.

The therapeutic implications of the reported role of PDGF-BB/PDGFR-β in the initiation of FDC-supported/lymphoid and the perpetuation of fibroblast-dominated/fibroid synovial pathotypes are substantial. In fact, earlier studies showed that PDGFR-β blockade and/or PDGF-BB neutralization inhibit synovial stromal cell migration, invadosome formation, and ameliorate disease progression in refractory RA (89–91). We reason that, PDGFR-β blockade could also abort early FDC differentiation and abrogate ELS formation in the RA synovium. Such early intervention may perhaps be therapeutically safer and more efficient than later targeting of immunologically active ELSs with sDMARDs and immunosuppressive biologics. Indeed, we have developed a single-chain variable fragment human Ab (scFv A7) that selectively targets pericytes in the RA synovium (92) and this Ab could be therapeutically exploited, in a bispecific format, to block PDGFR-β and inhibit pericyte-to-FDC differentiation.

Beyond its role in neuronal and cancer cell motility, the function of the CNA.42-binding protein, FBXO2, in human diseases, in general, and synovial disorders, in specific, is largely unknown. Nonetheless, murine studies with targeted deletion of FBXO2 showed progressive degeneration, loss of membrane integrity, and dysregulation of receptor localization and synaptic connectivity in the CNS (93, 94). Like the CNS, cells of the immune system communicate through progressive synaptic transmission, and it is plausible that FBXO2 plays a comparable role in FDCs by regulating surface retention of ICs and signal transmission in the FDC/B cell synapse.

In conclusion, PDGF-BB/PDGFR-β and TNF-α/LT-β reciprocally regulate RA synovial FDC development and pathotype differentiation. PDGF-BB induces early FDC differentiation from synovial pericytes and strongly correlates with fibroid synovitis. TNF-α/LT-β promotes full FDC maturation and is substantially associated with RA lymphoid pathotype. Therapeutic interference with PDGF-BB/PDGFR-β and TNF-α/LT-β mediated FDC differentiation and rheumatoid synovitis offers novel opportunities for patients suffering from aggressive/therapeutically resistant forms of RA.

## Data availability statement

The datasets presented in this study can be found in online repositories. The names of the repository/repositories and accession number(s) can be found below: https://www.ebi.ac.uk/arrayexpress/experiments/E-MTAB-6141/, E-MTAB-6141.

## Ethics statement

The studies involving human participants were reviewed and approved by Queen Mary University of London. The patients/participants provided their written informed consent to participate in this study.

## Author contributions

ME: conceptualization, study design, experiments, data acquisition, data analysis and interpretation, and manuscript preparation and revision. RE, NA, EP, MB, LF-J, and RH: experiments, data acquisition, and data analysis. ML: RNA-Seq data acquisition and analysis. CP: conceptualization, study design, interpretation of experimental results, and manuscript revision. All authors contributed to the article and approved the submitted version.

## Funding

The research leading to these results has received funding from the Barts Charity grant number MRC0177. The Pathobiology of early arthritis cohort (PEAC) was funded by the MRC grant 36661. Additional funding from MRC funded—Maximizing Therapeutic Utility for Rheumatoid Arthritis using genetic and genomic tissue responses to stratify medicines (MATURA)—Grant Ref: MR/K015346/1 and ARUK funded—Experimental Arthritis Treatment Center (EATC)—Grant Ref: 20022.

## Conflict of interest

The authors declare that the research was conducted in the absence of any commercial or financial relationships that could be construed as a potential conflict of interest.

## Publisher's note

All claims expressed in this article are solely those of the authors and do not necessarily represent those of their affiliated organizations, or those of the publisher, the editors and the reviewers. Any product that may be evaluated in this article, or claim that may be made by its manufacturer, is not guaranteed or endorsed by the publisher.
