# Supplementary material for: Follicular dendritic cell differentiation is associated with distinct synovial pathotype signatures in rheumatoid arthritis
Source: Front Med (Lausanne). 2022 Nov 16;9:1013660. doi: 10.3389/fmed.2022.1013660 (PMC9709129; doi:10.3389/fmed.2022.1013660)
Supplement: Supplementary file 1 [file Data_Sheet_1.PDF]

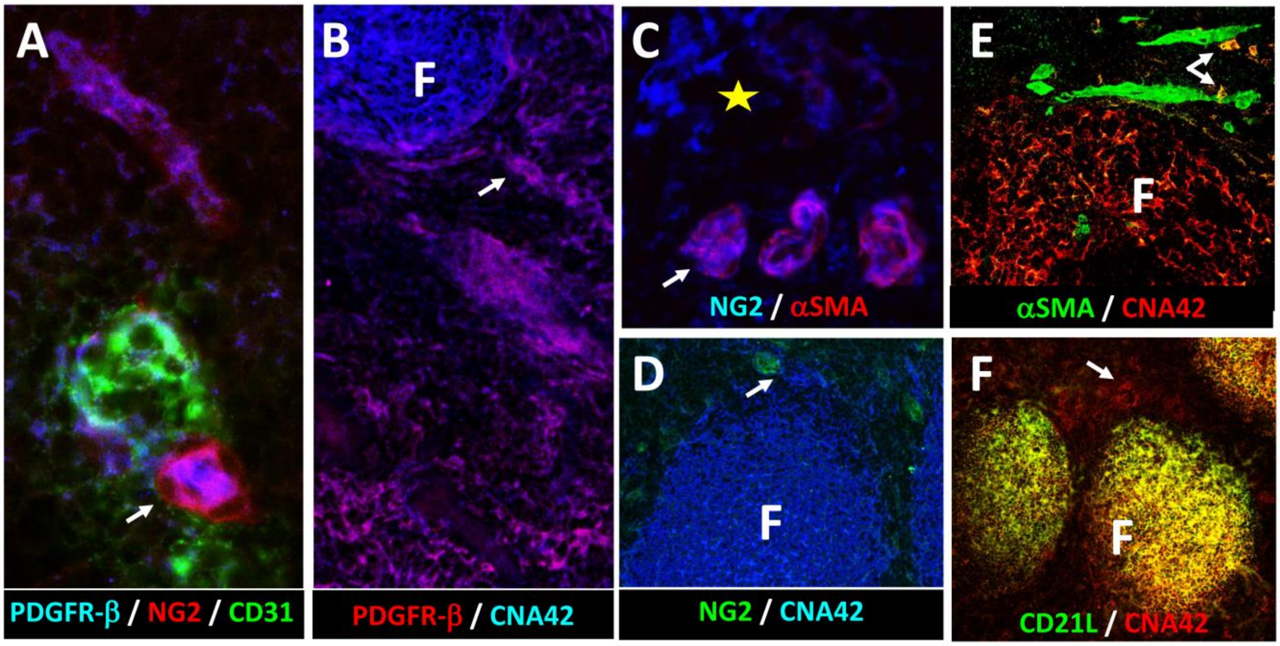

Supplementary Figure 1. Immunohistochemical colocalization of the FDC differentiation and maturation markers in the tonsils (A) The white arrow indicates colocalization of PDGFR-β (blue) with the pericyte marker NG2 (red) around the CD31$^+$ endothelium (green). (B) Outside the follicles (indicated by the letter F) the white arrow points at the colocalization of CNA.42$^+$ pre-FDCs (blue) with PDGFR-β (red). (C) The white arrow indicates the colocalization of αSMA (red) with NG2 (blue) on type 1 pericytes. αSMA$^-$/NG2$^+$ type 2 pericytes are located around the yellow star. (D) The white arrow indicates the colocalization of the pericyte marker NG2 (green) with the FDC marker CNA.42 (blue) in interfollicular areas. The follicles are demonstrated by 'F'. (E) The FDC marker CNA.42 (red) colocalizes with αSMA (green) in cells outside the mature follicles 'F' as indicated by the white arrows. (F) Mature (CD21L$^+$) and immature (CD21L$^-$) FDCs are both positive for CNA.42 (red). The colocalization of both markers in the follicles 'F' shows orange colour (CNA.42/red + CD21L/ green) while pre-FDCs in the interfollicular areas expressing CNA.42 only are red (white arrow).
